# Genomics of Urea Transport and Catabolism in Cyanobacteria: Biotechnological Implications

**DOI:** 10.3389/fmicb.2019.02052

**Published:** 2019-09-04

**Authors:** Théo Veaudor, Corinne Cassier-Chauvat, Franck Chauvat

**Affiliations:** Institute for Integrative Biology of the Cell (I2BC), CEA, CNRS, Univ Paris-Sud, Université Paris-Saclay, Gif-sur-Yvette, France

**Keywords:** urea transport and catabolism, urease, urea carboxylase, allophanate hydrolase, cyanobacteria

## Abstract

Cyanobacteria are widely-diverse prokaryotes that colonize our planet. They use solar energy to assimilate huge amounts of atmospheric CO_2_ and produce a large part of the biomass and oxygen that sustain most life forms. Cyanobacteria are therefore increasingly studied for basic research objectives, as well as for the photosynthetic production of chemicals with industrial interests. One potential approach to reduce the cost of future bioproduction processes is to couple them with wastewater treatment, often polluted with urea, which in any case is cheaper than nitrate. As of yet, however, research has mostly focused on a very small number of model cyanobacteria growing on nitrate. Thus, the genetic inventory of the cyanobacterial phylum is still insufficiently employed to meaningfully select the right host for the right purpose. This review reports what is known about urea transport and catabolism in cyanobacteria, and what can be inferred from the comparative analysis of the publicly available genome sequence of the 308 cyanobacteria. We found that most cyanobacteria mostly harbor the genes encoding the urea catabolytic enzymes urease (*ureABCDEFG*), but not systematically, together with the urea transport (*urtABCDE*). These findings are consistent with the capacity of the few tested cyanobacteria that grow on urea as the sole nitrogen source. They also indicate that urease is important for the detoxification of internally generated urea (re-cycling its carbon and nitrogen). In contrast, several cyanobacteria have *urtABCDE* but not *ureABCDEFG*, suggesting that *urtABCDE* could operate in the transport of not only urea but also of other nutrients. Only four cyanobacteria appeared to have the genes encoding the urea carboxylase (*uc*) and allophanate hydrolase (*ah*) enzymes that sequentially catabolize urea. Three of these cyanobacteria belongs to the genera Gloeobacter and Gloeomargarita that have likely diverged early from other cyanobacteria, suggesting that the urea carboxylase and allophanate hydrolase enzymes appeared in cyanobacteria before urease.

## Introduction

Cyanobacteria are ancient Gram-negative prokaryotes that perform the plant-like oxygen-evolving photosynthesis (here we consider the cyanobacterial phylum as consisting only of oxygenic phototrophs; Soo et al., 2017), which are regarded as the producers of our oxygenic atmosphere (Schopf, 2011), and the ancestor of plant chloroplasts (Archibald, 2009). In colonizing most waters (fresh, brackish and marine) soils and rocks of our planet, cyanobacteria have evolved as morphologically and metabolically widely-diverse microorganisms that are of high interest for basic and applied research, for a review see Cassier-Chauvat and Chauvat (2018). Attesting to their wide diversity, their genomes are widely diverse in size (and GC content, ranging from 30 to 60%), probably as the result of gain-and-loss of genes transferred by plasmids, insertion sequences and/or phages. Most cyanobacteria possess a single circular chromosome ranging from about 1.4 Mbp to about 9.0 Mbp in size and one to several plasmids (a few Kbp to several hundreds of Kbp in size). By contrast, a few marine cyanobacteria *(Prochlorococcus* and *Synechococcus*) have no plasmids, whereas *Cyanothece* ATCC 51142 possesses two chromosomes (one circular, 4.9 Mbp; and one linear, 0.4 Mbp) and four plasmids (ranging from 10 to 39 Kbp) (Shih et al., 2013). However, thus far research has mostly focused on a few model strains with well-established genetics and cyanobacterial diversity which has been insufficiently explored.

Collectively, cyanobacteria fix a huge amount of inorganic carbon (CO_2_) and nitrogen (N_2_, NH_4_, NO_2_, NO_3_ and urea) into an enormous biomass that supports a large part of the food chain. Hence, *Arthrospira platensis* strains have been used for centuries as food for animals and/or humans, and they are currently being tested as a way to replenish O_2_, recycle wastes (CO_2_ and urea) and provide food during long-term space missions (Verseux et al., 2016).

Cyanobacteria are also regarded as promising microbial factories for the ecological production of chemicals from solar energy, waters and atmospheric CO_2_ (for reviews see Knoot et al., 2018; Sun et al., 2018). To decrease the operating costs of such future cyanobacterial bio-refineries, it would be useful to develop a waste-to-biorefinery system that couples chemical production with waste-water treatment, i.e., feeding the cyanobacterial producer with organic pollutants, such as urea (Ramsundar et al., 2017) which is a cheaper nitrogen source than nitrate or ammonium and is often present in natural or waste waters.

Urea, the first organic molecule synthesized in the laboratory, is ubiquitous in nature (Carlini and Ligabue-Braun, 2016). In mammals, urea is the primary waste product of amino acid catabolism. It is distributed throughout the body and it is evacuated in urine (Rutherford, 2014). Thus, research is ongoing to recover urea (and phosphorus) from human urine (Zhang et al., 2014; Vasconcelos Fernandes et al., 2015). Urea is also present in natural waters due to its production by macro-fauna and benthic heterotrophic microorganisms, as well as its release by sediments from urea-fertilized agricultural soils (Glibert et al., 2006; Belisle et al., 2016) since about half of the nitrogen used for fertilization is applied as urea (Witte, 2011). In oceanic-estuarine waters, urea ranging from 1 nM to 50 μM can contribute to 50% or more of the total nitrogen used by cyanobacteria-rich phytoplanktonic communities (Solomon et al., 2010; Su et al., 2013). The diverse metabolic pathways of urea transport and decomposition may contribute to differences in the role that urea plays in the physiology and ecology of cyanobacteria, and in the role that each species plays in the biogeochemistry of urea.

Urea [(NH_2_)_2_CO] can be catabolized by several enzymes into NH_3_ (ammonia) and CO_2_ [(NH_2_)_2_CO + H_2_O ↔ CO_2_ + 2NH_3_], which can be re-injected into the cell metabolism. These ureolytic enzymes are, namely: urease (EC 3.5.1.5) and urea aminolyase (EC 6.3.4.6). They can be distinguished by their cofactor requirement (nickel for urease; ATP for urea amidolyase) as well as their inhibitor (hydroxyurea or acetohydroxamic acid for urease; avidin for amidolyase). In cyanobacteria, these inhibitors were used to show that *Anabaena cylindrica* ATCC 27899 possess an active urease but no urea amidolyase activity (Mackerras and Smith, 1986).

Urease (EC 3.5.1.5), also called urea aminohydrolase, is an important enzyme that plays a crucial role in various biological processes. It was the first enzyme to be crystallized and the first protein shown to contain nickel (for a review see Carlini and Ligabue-Braun, 2016). Urease is widespread in bacteria, fungi (except hemiascomyces), diatoms, and plants (Witte, 2011), but it is not present in green algae and animals. It catalyzes the ATP- and NAD(P)H-independent hydrolysis of urea into ammonia and carbamic acid (H_2_N–COOH), which is spontaneously hydrolyzed in carbonic acid (H_2_CO_3_) and a second ammonia molecule. These reactions are represented by the following equations (Carlini and Ligabue-Braun, 2016):

(NH)2CO2+HO2→NH+3HN2-COOH(ureaseactivity)

HN2-COOH+HO2→NH+3HCO23→NH+3HO2+CO(spontaneousreaction)2

NH+3HO2→NH+4+OH-

Under physiological conditions the proton of carbonic acid dissociates, and the ammonia molecules become protonated to form ammonium (NH_4_^+^) that increases the local pH. This can enable microorganisms to cope with acid challenges (Carlini and Ligabue-Braun, 2016). Hence, the pathogenic bacterium *Helicobacter pylori*, produces large amounts of urease (about 10% of its total proteins) to produce ammonia and to neutralize the acidic medium of the stomach to colonize it (this ammonia is toxic to host epithelial cells which significantly increases the risk of gastric ulcers and cancer). To persist in the gastric mucosa, *H. pylori* must also combat the host-produced reactive oxygen species. For this purpose, *H. pylori* uses the numerous methionine residues of its urease to quench the host oxidants; the resulting oxidized methionine residues being re-reduced by its methionine sulfoxide reductase (Schmalstig et al., 2018). Similarly, a urease-dependent alkalization of urine by *Proteus mirabilis* can lead to the formation of infection stones (ammonium magnesium phosphate or carbonate apatite) that contribute to the pyelonephritis (Rutherford, 2014).

Microbial ureases can also have beneficial roles for their hosts. The ureolytic bacteria thriving in the forestomach of ruminants cleaves animal-generated urea and releases ammonia that serves as the nitrogen source for the rumen microbiota, which plays a crucial role in the feeding of these animals (Carlini and Ligabue-Braun, 2016).

Plant ureases can have insecticidal effects (Witte, 2011). Upon plant ingestion by insects, the urease proteolysis catalyzed by the insect digestive enzyme releases peptides that can affect the contraction of insect muscles (Lopes et al., 2015).

In calcium-rich natural environments the increased pH and carbonate concentration caused by ureolytic microbes favors the formation and precipitation of calcium carbonate (CaCO_3_). This biomineralization process can be applied to the removal of calcium, heavy metals, and radionucleotides from water, as well as to the strengthening of soil, sand, stone, and cementitious materials. Many cyanobacteria of various genera have been reported to precipitate calcium carbonate as their metabolic product, thereby contributing to the formation of reservoir rocks like stromatolites and dolomites (Sarayu et al., 2014). The undeniable multifunctionality of ureases allows their inclusion in the moonlighting protein group (Carlini and Ligabue-Braun, 2016).

While most organisms catabolizing urea use a urease (EC 3.5.1.5) to break it down into ammonia and carbon dioxide, chlorophytes and some yeasts use the ATP- and biotin-dependent urea amidolyase enzyme (EC 3.5.1.4.5). This enzyme possesses two activities that can be exhibited by two different proteins (in prokaryotes and green algae): urea carboxylase (EC 6.3.4.6) and allophanate hydrolase (EC 3.5.1.54) (Kanamori et al., 2005). The urea carboxylase possesses two separate catalytic domains: the biotin carboxylase domain, where a tethered biotin cofactor is carboxylated by bicarbonate with concomitant ATP cleavage, and the carboxyl-transferase domain, where a carboxyl group is transferred from carboxybiotin to urea, forming allophanate. Subsequently, allophanate is hydrolyzed to ammonia and CO_2_ by allophanate hydrolase (Strope et al., 2011; Lin et al., 2016), as shown by the following equations:

(1) urea + ATP + HCO_3_ → urea-1-carboxylate (also called allophanate) + ADP + Pi
(2) allophanate + H_2_O → 2NH_3_ + 2CO_2_

It has been suggested that urea carboxylase and allophanate hydrolase co-evolved in bacteria and, following horizontal gene transfer, subsequently fused into a single urea amidolyase protein in fungi (Strope et al., 2011), not necessarily more active than the two enzymes urea carboxylase and allophanate hydrolase (Lin et al., 2016). Similar to bacterial urease, the yeast urea amidolyase can contribute to virulence and kidney disease pathogenesis of *Candida albicans* (Navarathna et al., 2012).

This review summarizes what is known about urea transport and catabolism in cyanobacteria, and what can be inferred from the comparative analysis of the publicly available genome sequence of 308 cyanobacteria.

## Results

### Cyanobacteria Have the Capability to Grow on Various Nitrogen Sources

Attesting their metabolic diversity, cyanobacteria exhibit different abilities to grow on various nitrogen sources (N_2_, NH_4_, NO_2_, NO_3_, and urea). The highly-abundant marine cyanobacteria of the genus *Prochlorococcus*, which employs a chlorophyll a/b light harvesting antenna instead of phycobilisomes of other cyanobacteria, cannot grow on NO_3_. The *Prochlorococcus* species can be classified into two major groups depending on their growth requirements. The low-chlorophyll b/a-containing *Prochlorococcus* ecotypes, which are adapted to high light and predominate in nutrient-depleted surface-waters of the open ocean, such as the strains MED4, MIT9215, MIT9312, MIT9401, and AS9601, grow well on recycled N sources NH_4_ and urea, but not on NO_2_ (Moore et al., 2002). In contrast, high-chlorophyll b/a (low-light adapted) ecotypes MIT9303, MIT9313, NATL1A, and NATL2A, which thrive in the deep euphotic zone, can grow on NH_4_, urea and NO_2_ which is often abundant at these depths (Moore et al., 2002). Thus, high-b/a and low-b/a Prochlorococcus partition the water column with respect to depth because of differences in not only their light utilization capabilities, but also their N utilization capabilities (ability to grow on NO_2_).

The other widely-abundant marine cyanobacteria of the genus *Synechococcus* that thrive in surface waters (such as strains PCC7002, WH7805, WH8102, WH8103) are able to grow on four N sources: NH_4_ (preferred substrate), urea (slight growth decrease), NO_3_, and NO_2_, except for *Synechococcus* MIT S9220 which cannot grow on NO_3_ (Sakamoto et al., 1998; Collier et al., 1999; Moore et al., 2002). These findings indicate that N, in addition to light, plays a critical role in determining the dynamics between the ecotypes of closely related marine *Prochlorococcus* and *Synechococcus* genera, and contributes to their stability in the world’s oceans (Moore et al., 2002).

Many urease-endowed cyanobacteria can grow on urea as the sole nitrogen source (Collier et al., 1999) but a high-concentration of urea and/or prolonged cultivation on urea (≥10 mM) can be toxic to cyanobacteria. This finding was shown with *Arthrospira* PCC 8005 (edible cyanobacterium, Deschoenmaeker et al., 2017), *Microcystis aeruginosa* (fresh water cyanobacterium, Wu et al., 2015), *Synechococcus* PCC 7002 (costal cyanobacterium, Sakamoto et al., 1998) and *Synechocystis* PCC 6803 (euryhaline cyanobacterium, Veaudor et al., 2018). The cell death and color change (from blue-green to yellowish) triggered by the prolonged growth on urea could be due to lipid peroxidation, a phenomenon that increases in parallel with cell death and pigment oxidation (Sakamoto et al., 1998). By contrast, urease defective mutants of *Synechococcus* PCC7002 and *Synechocystis* PCC 6803 (inactivation of the *ureC* gene, see below) were not killed by prolonged incubation in the presence of a high urea concentration, demonstrating that urea-consumption driven by urease can become toxic (Sakamoto et al., 1998; Veaudor et al., 2018). Furthermore, the (marine) *Synechococcus* WH7803 strain and the (freshwater) *Synechococcus* PCC7942 strain cannot grow on urea and neither have urease activity (Collier et al., 1999). This toxicity is not likely due to a urea-catabolism elicited modification of the pH, as these studies were carried out in the presence of pH buffers. Similarly, in plants, nitrogen nutrition based only on urea leads to a reduction in growth (Witte, 2011).

Urease activity appeared to be constitutive in the phylogenetically-distant cyanobacteria *Synechococcus* PCC 7002 cells (Ludwig and Bryant, 2012), *Synechocystis* PCC 6803 (Veaudor et al., 2018), and *Anabaena* PCC 7120 (Valladares et al., 2002). In contrast, the marine cyanobacteria *Synechococcus* WH7805 and *Synechococcus* WH8112 have a much higher urease activity when grown on NO_3_^–^ than on urea. In addition, *Synechococcus* WH7805 exhibits a twofold lower urease activity when grown on NH_4_^+^ compared to urea, whereas *Synechococcus* WH8112 has similar urease activities on NH_4_^+^ or urea. Urease expression increased in response to N deprivation in both of the *Prochlorococcus* strains MED4 and MIT9313 (Tolonen et al., 2006a). In cyanobacteria as diverse as *Anabaena* and *Prochlorococcus*, the urea transport genes are regulated by N availability *via* the global N transcription regulator NtcA (Valladares et al., 2002; Tolonen et al., 2006a), which also regulates urease activity in some cyanobacteria (Solomon et al., 2010).

### The Urease (*ureABCDEFG*) and Urea Transport (*urtABCDE*) Genes Are Widely Distributed in Cyanobacteria

In bacteria, urea penetrates in cells by passive diffusion or ATP-requiring uptake systems. Three types of urea transport systems have been described: the Yut protein in Yersinia, the UreI protein in Helicobacter and the UrtABCDE proteins in cyanobacteria (Valladares et al., 2002; Sachs et al., 2006). UrtA is the lipid-anchored urea binding protein; UrtB and UrtC are integral membrane proteins and UrtD and UrtE are ATP-binding proteins.

**FIGURE 1:**
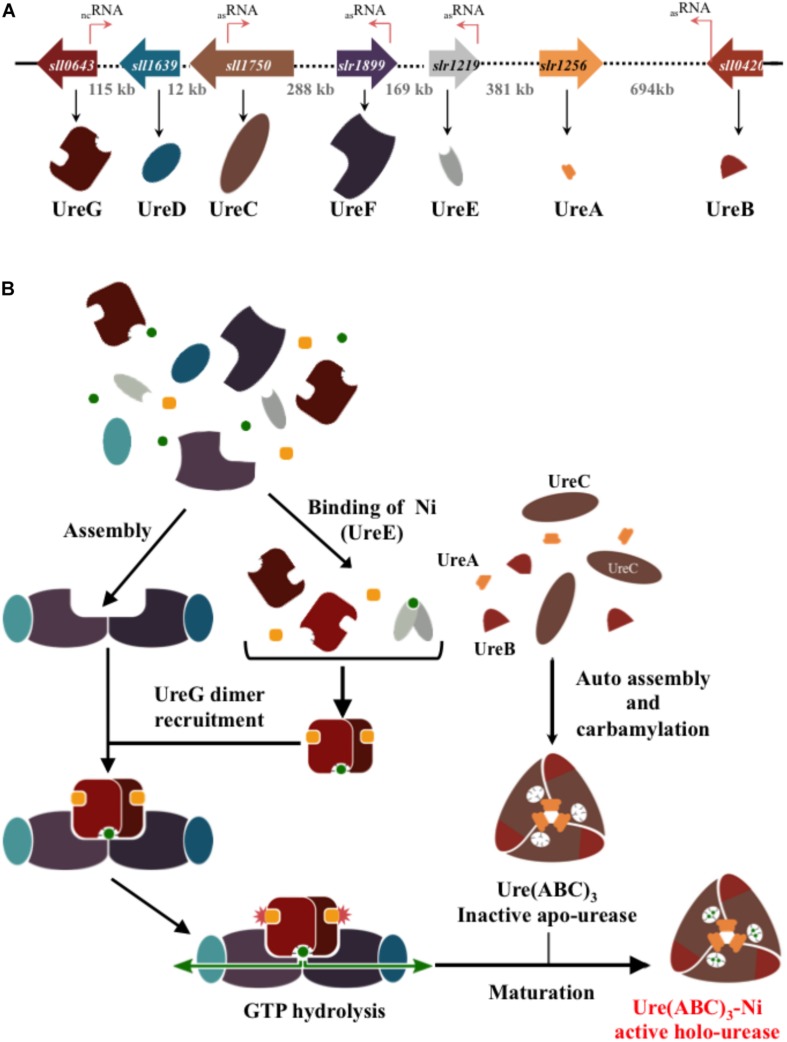
Schematic representation of the genes and proteins involved in the formation of the urease in the best-studied cyanobacterium *Synechocystis* PCC6803. **(A)** The genes encoding the urease catalytic subunits (UreABC) and the chaperones (UreDEFG) involved in its assembly are represented by large colored arrows indicating the direction of their transcription. The dotted lines and the numbers indicate the length of DNA (in Kb) separating the urease genes. The thin orange arrows stand for the small RNA (see cyanobase) of as yet unknow function. **(B)** Steps of the urease assembly and maturation process. The nickel ion and the guanoside-5′-triphosphate (GTP) are represented by green dots and orange squares, respectively.

Once inside the cells, urea can be catabolized by urease. Most bacterial urease is a trimer (UreABC)_3_ of two small (UreA and UreB) and one large (UreC, catalytic) subunits, while in plants the UreABC subunits are fused in a single protein (Witte, 2011). The urease enzyme complex is assembled by up to three (accessory) chaperone proteins (UreD, UreE, and UreF), and an intrinsically disordered (Palombo et al., 2017) GTPase (UreG) that transfers, likely with UreD (Farrugia et al., 2015), two nickel atoms into the urease metallocenter active site located in UreC (Figure 1). Mutation in either *ureD*, *ureE*, *ureF*, or *ureG* nearly abolish the activity of urease (Carter et al., 2009) The amino-acid sequences of the UreA, UreB, UreC, and to a slightly lesser extent of UreG, subunits are highly conserved, whereas UreD, UreE, and UreF sequences are more variable (Carter et al., 2009; Farrugia et al., 2015).

Cyanobacteria were shown to possess urease activity many years ago (Berns et al., 1966). Like other urease, the cyanobacterial enzyme depends on nickel (Mackerras and Smith, 1986; Collier et al., 1999) and can be inactivated by a mutation in *ureC* (Sakamoto et al., 1998; Collier et al., 1999; Wu et al., 2015; Deschoenmaeker et al., 2017; Veaudor et al., 2018). Furthermore, in *Anabaena* sp. PCC 7120 and *Synechocystis* PCC 6803 inactivation of *urtA*, *urtB*, and/or *urtE* strongly decreases (97–98%) urea uptake down to a level that likely corresponds to passive urea uptake (Valladares et al., 2002; Sachs et al., 2006).

Using a comparative genomic approach, we have analyzed the publicly available genome sequence of 308 cyanobacteria. We found that urea transport and utilization genes (Table 1) are unevenly distributed in cyanobacteria (Supplementary Table S1), in agreement with their wide genome diversity (Shih et al., 2013; Cassier-Chauvat et al., 2016).

**TABLE 1:** Reference of the *Synechocystis PCC 6803* (sll or slr) or *Gloeobacter violaceus* PCC 7421 (gll or glr) genes (from CyanoBase https://www.ncbi.nlm.nih.gov/protein) used to identify genes operating in urea acquisition and catabolism in other cyanobacteria.

**Gene**		
**name**	**Protein function/annotation**	**Gene ID**
	**Urease subunits**	
*ureA*	Urease subunit gamma	slr1256
*ureB*	Urease subunit beta	sll0420
*ureC*	Urease subunit alpha; catalytic subunit	sll1750
	**Maturation and assembly of urease**	
*ureD*	Urease accessory protein ureD (nickel ion binding)	sll1639
*ureE*	Urease accessory protein UreE	slr1219
*ureF*	Urease accessory protein UreF	slr1899
*ureG*	Urease accessory protein UreG parfois + UreH	sll0643
	**Urea transport**	
*urtA/amiC*	ABC high affinity urea uptake system substrate-binding UrtA	slr0447
*urtB/livH*	ABC high affinity urea uptake system permease UrtB/LivH	slr1200
*urtC*	ABC high affinity urea uptake system permease UrtC	slr1201
*urtD/livG*	ABC high affinity urea uptake system ATPase UrtD	sll0764
*urtE/braG*	UrtE branched-chain amino acid transport ATP-binding protein/livF	sll0374
	**Urea carboxylase and allophanate hydrolase**	
*uc*	Urea carboxylase/urea amidolase	gll0958
*ah*	Allophanate hydrolase	glr0961
*ucp1*	Urea carboxylase associated protein 1	gll0959
*ucp2*	Urea carboxylase associated protein 2	gll0960

The vast majority of the studied genomes (264 out of 308, i.e., 85%) possess all three genes encoding urease catalytic proteins (*ureABC*, Supplementary Table S2), mostly but not systematically accompanied with all five genes *ureDEFG* encoding urease accessory proteins (255 genomes, Supplementary Table S3). These findings show that urease is an abundant enzyme in cyanobacteria. The simultaneous presence (255 strains, Supplementary Table S3) or absence (40 strains, Supplementary Table S4) of all *ureABCDEFG* genes is consistent with the fact that both the urease catalytic proteins and the urease assembly proteins are essential to urease activity, as previously shown through a mutation in *ureC* or *ureG* (Sakamoto et al., 1998; Collier et al., 1999; Valladares et al., 2002; Veaudor et al., 2018). Thirteen cyanobacteria harbor an incomplete set of *ureABCDEFG* while *ureDEFG*, the urease chaperone genes, are more frequently absent than *ureABC*, the urease activity genes (Supplementary Table S5). This finding suggests that these cyanobacteria have either no active urease or they assemble it through an unknown process independent of some of the UreD, UreE, UreF, and UreG urease chaperones. The latter hypothesis is appealing in both *Aphanocapsa montana* BDHKU210001 and *Lyngbya confervoides* BDU141951 that possess a complete set of both *ureABC* (these genes are even duplicated in *Lyngbya confervoides* BDU141951) and *urtABCDE* (urea transport), in particular (Supplementary Table S5). In some cases, the absence of a gene might result from the fact that not all 308 cyanobacterial genomes are closed genomes. However, the lack of one or several of the *ureABCDEFG* genes has been observed in other (pathogenic) bacteria. *Helicobacter pylori* has only two urease-subunit genes, *ureA* and *ureB* (this *ureA* gene is a fusion of the *ureAB* genes occurring in other bacterial enzymes). These *H. pylori ureAB* genes are clustered with the following five downstream genes *ureI* (encoding a urea channel not encountered in other bacteria), *ureE*, *ureF*, *ureG*, and *ureH* (UreH is a nickel permease homologous to the UreD subunit of other bacteria). Thus, *H. pylori* has a UreH/UreF/UreG complex, not a UreD/UreF/UreG complex (Fong et al., 2013). *Y. pestis* harbors a complete urease locus (*ureABC*) and four accessory (*ureEFGD*) genes, but it has no urease activity because the nickel-incorporation gene *ureD* is disrupted (Carlini and Ligabue-Braun, 2016). Similarly, *Bacillus subtilis* has no urease accessory genes, suggesting that accessory proteins are not always required for *in vivo* urease activation or that genetically distinct cellular maturation factors are utilized in some cases (Carter et al., 2009).

Two lines of evidence showed that urease transport genes are also widely distributed in cyanobacteria. First, 235 strains harbor all urtABCDE genes, which are duplicated in the two strains of the genus *Acaryochloris* (Supplementary Table S6). Second, among the 255 cyanobacteria that possess all urease activity genes *ureABCDEFG* (Supplementary Table S3) 225 strains also have all urea transport genes *urtABCDE* (Supplementary Table S7). For example, among the 45 *Prochlorococcus* strains presently studied, 34 have *ureABCDEFG* and *urtABCDE*, including the species MED4, MIT9215, MIT9312, MIT9401, MIT9303, MIT9313, NATL1A, and NATL2A, which grow well on urea (Moore et al., 2002). The other 11 *Prochlorococcus* strains lack both *ureABCDEFG* and *urtABCDE* (Supplementary Table S4), as mentioned earlier for MIT9211, MIT8515, and CCMP1375, the growth of which was not tested on urea (Solomon et al., 2010). Furthermore, 235 cyanobacteria have all *urtABCDE* genes.

A minority of the cyanobacteria endowed with a complete set of urease genes *ureABCDEFG* possess no urea transport genes *urtABCDE* (23 strains, Supplementary Table S8). This observation suggests that the urease of these cyanobacteria likely operates in the detoxification of internally generated urea (recycling of nitrogen lost during the urea-generating catabolism of arginine). It is also possible that urea diffuses through aquaporins as observed in other organisms (Li and Wang, 2014). Conversely, several cyanobacteria harbor *urtABCDE* but an incomplete set of *ureABCDEFG* (10 strains, Supplementary Table S5), suggesting that *urtABCDE* could operate in the transport of not only urea but also other nutrients.

### Occurrence of Duplication of Either Urea Transport (*urtABCDE*) or Urease Activity (*ureABCDEFG*) Genes but Not Both

Twenty-nine cyanobacteria harbor two copies of one or several genes *ureABC* (urease activity)*, ureDEFG* (urease assembly) and/or *urtABCDE* (urea transport). In 13 cases, the duplication concerned one or two, but not all three sets of *ureABC, ureDEFG* and *urtABCDE* (Supplementary Table S9). Seven cyanobacteria possess two copies of *ureABC*, three strains have two copies of *ureDEFG*, while six strains harbor two copies of *urtABCDE*. Two cyanobacteria possess two copies of all urease assembly and activity genes *ureABCDEFG* (*Chamaesiphon minutus* PCC 6605 and *Mastigocoleus testarum* BC00), while one strain has two copies of both *ureABC* and *urtABCDE* (Xenococcus sp. PCC 7305). By contrast, we found no cyanobacterium with a duplication of *ureDEFG* and *urtABCDE* (Supplementary Table S9). In the future, it will be interesting to study whether the products encoded by these duplicated genes have distinct biochemical properties or are differently regulated.

The occurrence of multiple copies of urease genes is not unprecedented in prokaryotes. For instance, the beta-proteobacterium *Nitrosospira* strain NpAV possesses two copies of *ureC* (Koper et al., 2004; Carter et al., 2009). Furthermore, three *Helicobacter species* (i.e., *H. mustelae*, *H. acinonychis*, and *H. felis*) harbor two sets of urease genes (Fong et al., 2013). The first set contains the complete urease gene cluster (*ureA1B1EFGH*) while the second set *ureA2B2* (50% identical to *ureA1* and *ureB1*, respectively) encodes the UreA2B2 enzyme that does not require urease accessory proteins to be active. The *ureA1B1EFGH* cluster encoding the Ni-containing urease is induced by nickel ions while the *ureA2B2* cluster encoding an iron-containing enzyme is up regulated by Fe (it is downregulated by nickel). These findings are consistent with the observation that these *Helicobacter* species are associated with carnivores that eat an iron-rich food depleted in nickel (Carter et al., 2009).

We found no duplication of all three gene-sets *ureABC, ureDEFG*, and *urtABCDE* (Supplementary Table S9), suggesting that a very high transport and catabolism of urea would be toxic. This hypothesis is consistent with the findings that phylogenetically-distant cyanobacteria were killed by a prolonged growth on high urea concentration, namely Arthrospira PCC 8005 (Deschoenmaeker et al., 2017), *Microcystis aeruginosa* (Wu et al., 2015), *Synechococcus* PCC 7002 (Sakamoto et al., 1998), and *Synechocystis* PCC 6803 (Veaudor et al., 2018).

### Distribution of the Genes Encoding the Other Urea Catabolytic Enzymes: Urea Carboxylase and Allophanate Hydrolase Genes Are Less Frequent Than the Urease Genes

Several lines of evidence suggest that the urea carboxylase and/or allophanate hydrolase enzymes have little importance for the photoautotrophic metabolism of cyanobacteria.

First, among the 308 cyanobacteria presently studied 237 have a complete set of urease genes *ureABCDEFG* whereas they lack one or both the *uc* and *ah* genes encoding the urea carboxylase and allophanate hydrolase (Supplementary Table S10). Second, a few cyanobacteria have *uc* (19 strains) and/or *ah* (13 strains), while only nine cyanobacteria possess both *uc* and *ah* (Supplementary Table S11), which together, could allow a urease-independent urea catabolysis, as shown in other organisms (Strope et al., 2011; Lin et al., 2016). Third, four of these nine cyanobacteria endowed with *uc* and *ah* are actually devoid of the *ureABCDEFG* urease genes, namely *Calothrix* sp. PCC 7507, *Gloeomargarita lithophora* D10, *Gloeobacter kilaueensis* JS1, and *Gloeobacter violaceus* PCC 7421 (Supplementary Table S11). It will be interesting in the future to test if these cyanobacteria are able to grow on urea as the sole nitrogen source to verify whether their urea carboxylase and allophanate hydrolase are truly active. Because, *Gloeobacter* and *Gloeomargarita* are regarded to have diverged early from other cyanobacteria (de Vries and Archibald, 2017; Ponce-Toledo et al., 2017), it is possible the urea carboxylase and allophanate hydrolase enzymes appeared in cyanobacteria before urease. Furthermore, interestingly, *Calothrix* sp. PCC 7507, *Gloeomargarita lithophora* D10, *Gloeobacter kilaueensis* JS1, and *Gloeobacter violaceus* PCC 7421 not only lack the genes encoding the Ni-containing urease but also the genes encoding the Ni-Fe hydrogenase. This finding suggests that these cyanobacteria may live in nickel poor environments.

### Few Cyanobacteria Possess the Complete Panoply of Urea Transport (*urtABCDE*) and Catabolism (*ureABCDEFG*, *uc*, and *ah*) Genes

Only five cyanobacterial strains possess the complete panoply of urea transport (*urtABCDE*) and catabolism (*ureABCDEFG*, *uc*, and *ah*) genes (Supplementary Table S12), namely *Cyanothece* PCC7425, *Microcoleus vaginatus* FGP-2, *Microcoleus vaginatus* PCC 9802, and S*ynechococcus* PCC 7502. This finding suggests that these five cyanobacteria growing in their natural environment frequently uses urea as a nitrogen source. We have verified that *Cyanothece* PCC 7425 can grow not only on nitrate and ammonium, but also on urea as the sole nitrogen source.

### Several Cyanobacterial Genera Display a Highly Heterogeneous Panel of Urea Acquisition and Catabolism Genes

**FIGURE 2:**
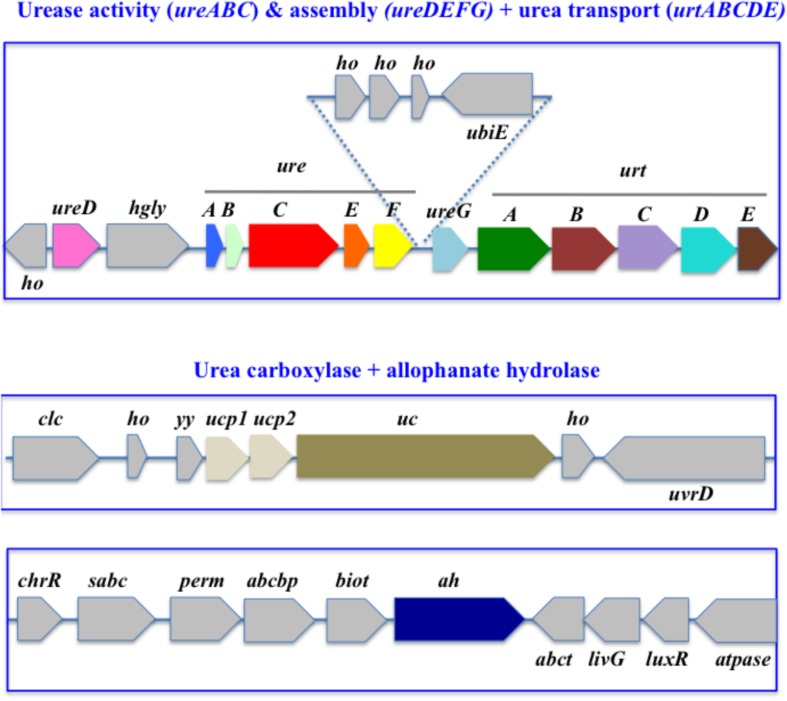
Organization of the urea acquisition and catabolism genes in *Cyanothece* PCC 7425. The genes are shown as colored boxes pointing in the direction of their transcription. Gray boxes correspond to genes not known to be involved in urea metabolism or transport. *abcbp*, ABC transporter ATP-binding protein; *biot*, biotin-[acetyl-CoA-carboxylase] synthetase; *chrR*, anti-ECF regulator; *clc*, chloride channel core; ubiE, ubiquinone biosynthesis protein; *ho*, hypothetical protein; *sabc*, sulfonate binding ABC transporter protein; *ucp1*, urea carboxylase associated protein 1; *ucp2*, urea carboxylase associated protein 2; *uvrD*, DNA helicase; *yy*, YKKC-YXXD leader in MBGD; *perm*, ABC transporter permease; *abct*, amino acid ABC transporter ATPase; *livG*, leucine/isoleucine/valine transporter ATP-binding subunit; *luxR*, LuxR family transcriptional regulator; atpase, ATPase.

Among the four *Calothrix* strains, three possess the *ureABCDEFG* genes but neither *urtABCDE*, nor *uc* and *ah*, whereas Calothrix PCC 7507 lacks *ureABCDEFG* and *urtABCDE* but possess both *uc* and *ah* (Supplementary Table S13). The eight studied *Cyanothece* strains also have different gene panoplies. *Cyanothece* PCC 7425 phylogenetically distant for the other *Cyanothece* has all urea transport and catabolism genes (*ureABCDEFG*, *urtABCDE*, *uc*, and *ah*, see Figure 2), whereas the two *Cyanothece* strains PCC 7424 and PCC 7822 (Supplementary Table S1 and Supplementary Figure S1) possess *ureABCDEFG* and *urtABCDE*, but neither *uc* nor *ah*, and the five other strains ATCC 51472, ATCC 51142, CCY 0110, PCC 8801, and PCC 8802 lack all *ureABCDEFG*, *urtABCDE*, *uc* and *ah* genes (Supplementary Tables S1, S4). These observations are consistent with the findings that *Cyanothece* ATCC51142, PCC 8801, and PCC 8802, which fix atmospheric N_2_ in aerobiosis, are less dependent of an organic nitrogen source than *Cyanothece* PCC 7425, which fix N_2_ only in anaerobiosis (Bandyopadhyay et al., 2011).

The 12 *Leptolyngbya* strains display a different panel of the urea acquisition and catabolism, in agreement with phylogenetic analysis showing that *Leptolyngbya* strains are found in different clusters. Eight strains have all the *ureABCDEFG* and *urtABCDE* genes, but one of them has the *uc* gene but not *ah* (*Leptolyngbya boryana* PCC 6306) whereas two of them have *ah*, but not *uc* (*Leptolyngbya* sp. PCC 7375 and *Leptolyngbya* sp. Heron Island J). Furthermore, two of these eight strains harboring the *ureABCDEFG* and *urtABCDE* genes have a duplication of all the *urtABCDE* genes and posess *uc* (*Leptolyngbya* sp. NIES-2104 and *Leptolyngbya* sp. NIES-3755), whereas one strain has two copies of *urtAB* genes but possess neither *uc* nor *ah* (*Leptolyngbya* sp. PCC 6406). *Leptolyngbya valderiana* BDU 20041 has neither *ureFG* nor *urtABCDE* genes. Furthermore, among the 45 *Prochlorococcus* species analyzed, 34 strains have *ureABCDEFG* and *urtABCDE*, whereas 11 strains lack both *ureABCDEFG* and *urtABCDE* (Supplementary Table S14). In contrast the *Microcystis* genera is homogenous since all 14 *Microcystis aeruginosa* strains possess all *ureABCDEFG* and *urtABCDE* genes, but neither *uc* nor *ah*.

### Several Cyanobacteria Lack the Whole Panoply of Urea Transport and Catabolism Genes

Thirty-six cyanobacteria lack the complete set of urea transport (*urtABCDE*) and catabolism (*ureABCDEFG*, *uc*, and *ah*) genes (Supplementary Table S14). This is the case of all four strains of the genus *Crocosphaera watsonii* (marine unicellular diazotrophic cyanobacteria) and all four strains of the genus *Gastranaerophilaceae* (belonging to the new phylum of Melainabacteria that are not able to perform photosynthesis.

The same is true for the symbiotic (marine) cyanobacterium UCYN-A, in agreement with the fact that it possesses the smallest genome (1.44 Mb), and for the two strains of *Richelia intracellularis*. Similarly, 11 strains of the *Prochlorococcus* genus, which is known to possess a small-genome, are totally devoid of the urea transport (*urtABCDE*) and catabolism (*ureABCDEFG*, *uc*, and *ah*) genes. By contrast, the 34 other *Prochlorococcus* strains have *ureABCDEFG* and *urtABCDE* (but neither *uc* nor *ah*). Also similarly, five *Synechococcus* strains lacks all urea transport and catabolism genes, including the two closely-related models *Synechococcus elongatus* PCC7942 and *Synechococcus elongatus* PCC6301 and the fast-growing strain *Synechococcus elongatus* UTEX 2973 (doubling time as short as 1.5–1.9 h) that are closely related (there are only 55 single nucleotide differences separating the two strains (Ungerer et al., 2018; Supplementary Table S15 model strains).

### Genomic Context of Urea Transport and Catabolism Genes in Model Cyanobacteria: The Urea Transport Genes (*urtABCDE*) Are Clustered Whereas the Urease Genes (*ureABCDEFG*) Are Often Scattered

In bacteria, the three urease structural genes *ureABC* are often clustered with those genes encoding urease associated proteins *ureCDEFG*, but their number and order differ among species (Carter et al., 2009). In *K. aerogenes*, *ureABC* are flanked by *ureDEFG* in a *ureDABCEFG* gene cluster. The same gene order, *ureDABCEFG*, occurs in the Beta-proteobacterium *Nitrosospira* NpAV and the Gamma-proteobacterium *Nitrosococcus oceani* (Koper et al., 2004). By contrast, many bacteria position *ureD* after *ureG*, like that occurring in the thermophilic *Bacillus* sp. TB-90 (Carter et al., 2009).

We performed a gene neighborhood survey of the genes operating in urea acquisition and assimilation in cyanobacteria emphasizing on phylogenetically distant model cyanobacteria (Supplementary Table S15). We focused our attention on several phylogenetically-distant cyanobacteria (Supplementary Table S16) because they are presently well studied thanks to their powerful genetics (for example *Synechocystis* PCC 6803, *Synechococcus* PCC 7002, and *Synechococcus* PCC 7942). Furthermore, they should also be increasingly investigated in the near future because of their interesting natural properties (Supplementary Table S16) and the likely possibility that they could be manipulated with a broad-host-range of RSF1010 plasmids that have been shown to replicate in various cyanobacteria since first being reported (Marraccini et al., 1993; Mühlenhoff and Chauvat, 1996; Tolonen et al., 2006a, b; Araki et al., 2013; Taton et al., 2014).

**FIGURE 3:**
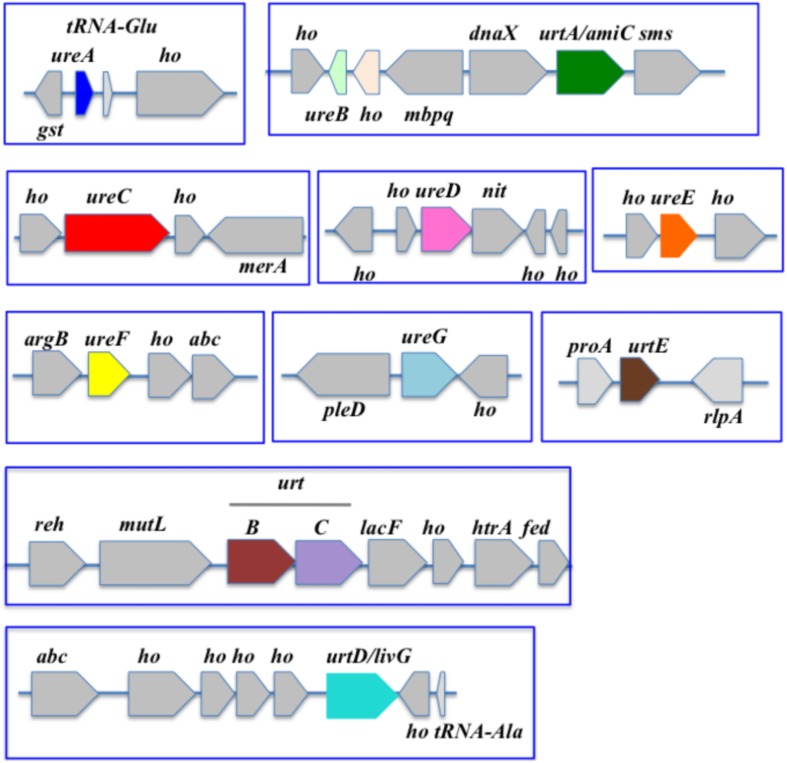
Organization of the urea acquisition and catabolism genes in *Synechocystis* PCC 6803. The genes are shown as colored boxes pointing in the direction of their transcription. Gray boxes correspond to genes not known to be involved in urea metabolism or transport. *abc*, ABC transporter; *argB*, acetylglutamate kinase; *abc*, ABC transporter; *dnaX*, DNA polymerase III subunit delta; *fed*, ferredoxin; *gst, glutathione-S-transferase; ho*, hypothetical protein; *htrA*, serine protease; *lacF*, lactose ABC transporter permease; *mbpq*, methyl-6-phytyl 1,4-hydroquinone methyltransferase; *merA*, mercuric reductase; *mutL*, DNA mismatch repair; *nit*, nitrilase; *pleD*, pleD protein; *proA*, gamma-glutamyl phosphate reductase; *radA* homolog; *reh*, rehydrin; *rlpA*, rare lipoprotein A; sms, DNA repair protein.

**FIGURE 4:**
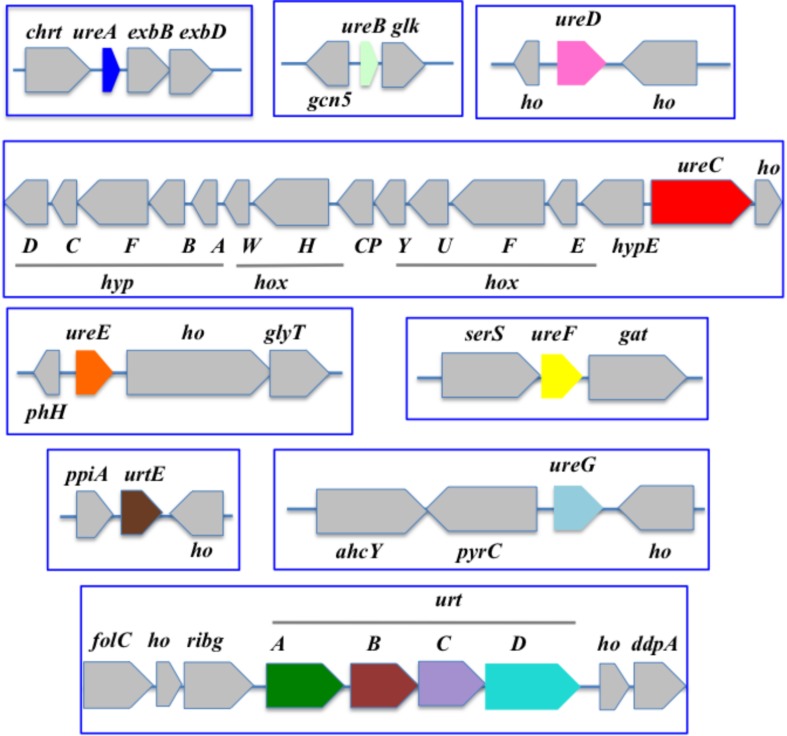
Organization of the urea acquisition and catabolism genes in *Synechococcus* PCC 7002. The genes are shown as colored boxes pointing in the direction of their transcription. Gray boxes correspond to genes not known to be involved in urea metabolism or transport. *ahcY*, adenosyl homocysteinase*; chrt*, chromate transport; *ddpA*, peptide ABC transporter substrate binding protein; *exbB* and *exbD*, biopolymer transport; *folC*, folypolyglytamte; *gat*, glutamine amidotransferase. *gcn5*, N-acetyltransferase GCN5; *glk*, glucokinase; *glyT*, glycosyl transferase; *ho*, hypothetical protein; *hoxEFGHU*, Ni-Fe hydrogenase subunits; *hoxW*, HoxH-specific protease; *hypABCDEF*, Ni-Fe hydrogenase chaperone subunits; *phh*, phosphohydrolase; *ppia*, peptidylprolyl isomerase A; *pyrC*, dihydroorotase*; ribg*, ADP-ribosylglycohydrolase; *serS*, serine-tRNA ligase synthase.

Our gene neighborhood survey revealed that these selected cyanobacteria harbor diverse panoplies of urea acquisition and utilization genes that define five groups. The first group comprises 13 cyanobacteria, including the extensively-studied species *Synechocystis* PCC6803 (Figure 3) and *Synechococcus* PCC 7002 (Figure 4), which possess a single copy of the *ureABCDEFG* and *urtABCDE*, but neither *uc* nor *ah* (Supplementary Table S15 and Supplementary Figure S1). The second group of cyanobacteria (five species including the well-studied strain *Synechococcus* PCC 7942) has no urea acquisition and catabolic genes. The third, fourth and fifth groups are each defined by a single model cyanobacterium, as follows. *Cyanothece* PCC 7425 (Figure 2) possesses the complete panoply of the studied genes (Supplementary Table S12). *Gloeobacter violaceus* PCC 7421 has only *uc* + *ah*, the importance of which can be studied in this host or in *Cyanothece* PCC 7425. *Acaryochloris marina* MBIC11017 possesses *ureABCDEFG* + a duplicated copy of *urtABCDE* (Supplementary Figure S1).

Our gene neighborhood survey revealed that *ureA, ureB*, and *ureC* were often contiguous, as were *ureE, ureF*, and *ureG*. Furthermore urease genes are often clustered with those genes encoding the ATP-dependent uptake system (*urtABCDE*) (Valladares et al., 2002; Wu et al., 2015).

A closer look at the gene organization of these cyanobacteria shows the following findings. The chlorophyll d-containing symbiotic cyanobacterium *Acaryochloris marina* MBIC11017 has the particularity of having two spatially distant *urtABCDE* clusters (Supplementary Figure S1). One of them is located downstream of the *ureFG* cluster. The *ureE* gene is located far away, including from the locus encompassing the *ureDA* upstream of *ureB* and *ureC* in that order.

In the edible (Deschoenmaeker et al., 2017) cyanobacterium *Arthrospira* PCC 8005 (Supplementary Figure S1), *urtABCDE* and *ureDABC* are displayed, in that order, in two non-neighboring clusters, away from *ureEF* and *ureG*.

**FIGURE 5:**
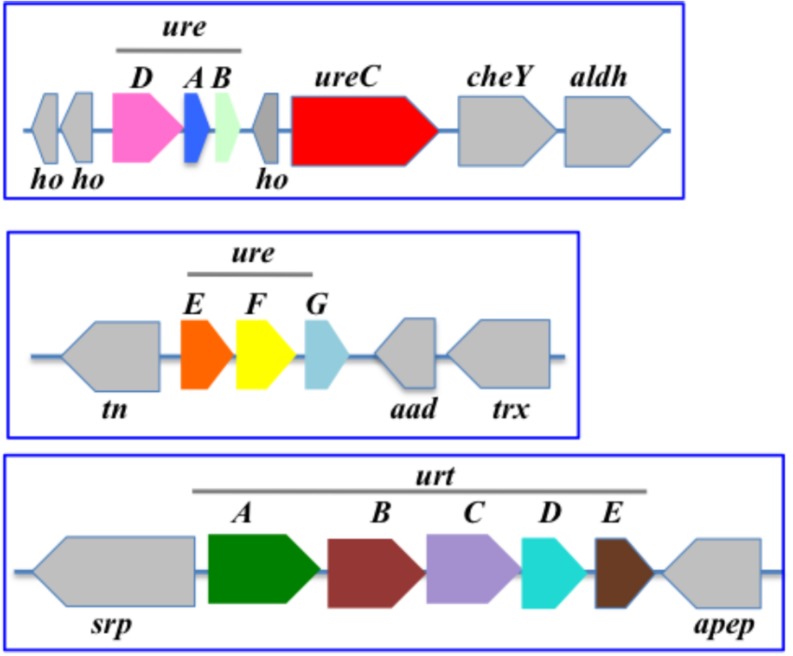
Organization of the urea acquisition and catabolism genes in *Nostoc* PCC 7120. The genes are shown as colored boxes pointing in the direction of their transcription. Gray boxes correspond to genes not known to be involved in urea metabolism or transport. Each blue box corresponds to a genomic region. *aad*, acetoacetate decarboxylase; *aldH*, aldehyde dehydrogenase; *apep, a*minopeptidase; *ho*, hypothetical protein; *cheY*, chemotaxis protein CheY; *tn*, tranposase; *srp*, signal recognition particle; *trx*, thioredoxin.

In *Nostoc* (*Anabaena*) PCC7120 (Figure 5), the urea transport genes (*urtABCDE*) are clustered in that order, whereas the urease (*ureABCDEFG*) is displayed in two loci. One locus comprises the cluster *ureDAB* and *ureC* separated by an unknown gene, while the other locus contains *ureEFG* in that order, as previously observed (Valladares et al., 2002).

In *Cyanothece* PCC 7425, which has the complete panoply of urea transport and catabolism genes, the urease (*ureABCDEFG*) and urea transport (*urtABCDE*) genes are located in the same chromosomal region (Figure 2) where *ureD* precedes the two clusters *ureABCDEF* and *ureG-urtABCDE*. In contrast, the *uc* and *ah* genes are not close to each other. Interestingly, the *uc* genes is clustered with two genes, *ucp1* and *ucp2*, which presumably code for two urea carboxylase associated proteins. A similar situation occurs in several other cyanobacteria such as *Chamaesiphon minutus* PCC 6605, *Gloeocapsa* PCC 7428, *Oscillatoria* PCC 6505, *Oscillatoria nigro viridis* PCC 7112, *Microcoleus vaginatus* FGP-2, *Pseudanabaena biceps* PCC7429, and *Synechococcus* PCC7502.

In *Cyanothece* PCC 7822, the urease (*ureABCDEFG*) and urea transport (*urtABCDE*) genes define two non-neighboring clusters, *ureABGDEFC* and *urt ABCDE* (Supplementary Figure S1).

In *Gloeobacter violaceus* PCC 7421, which has neither *ureABCDEFG* nor *urtABCDE*, the *ah* and *uc* genes are located in the same region but in opposite directions (Supplementary Figure S1).

In the well-studied heterocyst-forming filamentous strain *Anabaena* PCC 7120, *ureDAB* are clustered upstream of *ureC*, far away from both the *ureEFG* and the *urtABCDE* clusters (Figure 5).

In the marine *Prochloroccus* strains MED4 and MIT9313, the *ure* and *urt* genes are located in the same locus comprising two opposite clusters *urtEDCBAGFE* and *ureDABC* (Supplementary Figure S1).

In the marine strain *Synechococcus* WH8102 (Supplementary Figure S1), the *ure* and *urt* genes are located mostly in one locus comprising two opposite clusters *urtEDCBAureGFE* and *ureDABC* (as observed in the *Prochloroccus* strains MED4 and MIT9313) far away from *urtA2* the second copy of *urtA* (Supplementary Figure S1).

In the costal strain *Synechococcus* PCC7002 (Figure 4), the *urt* genes are displayed in two non-neighboring regions harboring the *urtABCD* cluster and *urtE*, respectively. In contrast, the *ure* genes are scattered in different loci. One of them harbors *ureC* in an opposite direction from the clustered genes *hox* and *hyp* encoding hydrogenase, the activity of which requires Ni like urease. It is possible that the proximity of the genes encoding Ni-dependent enzymes facilitates their Ni-dependent expression.

In *Synechocystis* PCC 6803, the *ureABCDEFG* and *urtABCDE* genes all are completely scattered (Figure 3).

Finally, in *Thermosynechococcus elongatus BP1*, the urea transport genes (*urtABCDE*) are clustered in that order, whereas the urease (*ureABCDEFG*) are scattered (Supplementary Figure S1).

## Discussion

Cyanobacteria are widely diverse photosynthetic microorganisms that play a crucial role for the biosphere (production of biomass and oxygen for the biosphere) and have great biotechnological interests (photoproduction of chemicals). As of yet, however, research has mostly focused on few model strains with well-established genetics, while cyanobacterial diversity is insufficiently explored. Furthermore, most studies on cyanobacteria are carried out with cells growing on nitrate (NO_3_) while we show in this review that most cyanobacteria have the potential to grow on urea [(NH_2_)_2_CO] as the nitrogen source. This finding is important because urea is cheaper than nitrate or ammonium, and it is often present in natural waters because it is produced by heterotrophic organisms and it is released by the sediments of urea-fertilized agricultural soils (Glibert et al., 2006; Belisle et al., 2016). Thus, in the future it will be interesting to use cyanobacteria to couple the photosynthetic production of chemicals and the removal of the urea pollutant, in economically viable waste-to-biorefinery industrial systems.

In this study, we report what is known about urea transport and catabolism in cyanobacteria, and what can be inferred from the comparative analysis of the publicly available genome sequence of 308 cyanobacteria.

We show that most cyanobacteria possess the genes encoding one or several enzymes, urease, urea carboxylase and allophanate hydrolase, which catabolize urea [(NH_2_)_2_CO] into NH_3_ (ammonia) and CO_2_ that can be re-injected into the cell metabolism.

Urease is a very frequent enzyme in cyanobacteria. The vast majority of the 308 studied genomes (264 out of 308, i.e., 85%) possess all three genes encoding urease catalytic proteins (*ureABC*, Supplementary Table S2), mostly accompanied with all four genes *ureDEFG* encoding urease accessory proteins (255 genomes, Supplementary Table S3). These findings are consistent with the fact that both the urease catalytic and assembly proteins are essential to urease activity, as previously shown through mutations in *ureC* or *ureG* (Sakamoto et al., 1998; Collier et al., 1999; Valladares et al., 2002; Veaudor et al., 2018). A large number of these 255 cyanobacteria possessing *ureABCDEFG* (Supplementary Table S3) also have all urea transport genes *urtABCDE* (225 strains, Supplementary Table S7), suggesting that they grow in urea-containing environments. Interestingly, we found that the urtABCDE genes are frequently clustered, whereas *ureABCDEFG* are often scattered (Figures 2–5 and Supplementary Figure S1). A minority of cyanobacteria possessing all urease genes (*ureABCDEFG)* lack all urea transport genes *urtABCDE* (23 strains, Supplementary Table S8) thereby suggesting that their urease operates in the detoxification of internally-generated urea (for example by the catabolism of arginine). Conversely, several cyanobacteria harboring *urtABCDE* lack one or several *ureABCDEFG* genes (10 strains, Supplementary Table S5), suggesting that *urtABCDE* could operate in the transport of not only urea but also of other nutrients.

Two lines of evidence suggest that the urea carboxylase and/or allophanate hydrolase enzymes, encoded by the *uc* and *ah* genes, have less importance than urease for the metabolism of cyanobacteria. First, only five of the 308 cyanobacteria presently studied have the complete panoply of urea transport (*urtABCDE*) and catabolism (*ureABCDEFG*, *uc* and *ah*) genes. Second, only four cyanobacteria that possess both the *uc* and *ah* genes (Supplementary Table S11) presumably involved in urea catabolysis are actually lacking the *ureABCDEFG* urease genes. These cyanobacteria are *Calothrix* PCC 7507, *Gloeomargarita lithophora* D10, *Gloeobacter kilaueensis* JS1, *Gloeobacter violaceus* PCC 7421 (Supplementary Table S11). It will be interesting to assay if these cyanobacteria can really grow on urea as the sole nitrogen source, to test whether the *uc* and *ah* genes truly operates in urea catabolism. Interestingly, these four cyanobacteria that lack the genes encoding the Ni-requiring urease enzyme also lack the genes encoding the Ni-Fe hydrogenase, suggesting that they may live in nickel-poor environments. Because the cyanobacteria of the genera *Gloeobacter* and *Gloeomargarita* are regarded as having diverged early from other cyanobacteria (de Vries and Archibald, 2017; Ponce-Toledo et al., 2017) it is possible that the urea carboxylase and allophanate hydrolase enzymes appeared in cyanobacteria before urease, and were subsequently lost by various cyanobacteria, most of which possess urease genes, to catabolize urea by the ATP-independent urease instead of the ATP- and HCO3-consuming enzymes urea carboxylase and allophanate hydrolase. This assumption is supported by the phylogenetic analysis of the distribution of the genes encoding the urease (ureC subunit, panel **A**), allophanate hydrolase (**B**) and urea carboxylase (**C**) proteins in various organisms (Supplementary Figure S2). Among the 264 cyanobacterial UreC amino-acids sequences considered in this study, 39 were selected so as to maximize the coverage and representation of all five sub-sections of the cyanobacterial phylum proposed by other workers (Shih et al., 2013). All these genes appeared to be distributed in the cyanobacterial clade, likely ruling out horizontal gene transfer events, which if occurs, would blur their distribution. Similarly, all cyanobacterial *uc* (19) and *ah* (13) genes were found to be distributed within the cyanobacterial clade, likely in absence of horizontal-gene-transfer introduction of foreign *uc* and/or *ah* genes into some cyanobacteria. However, one of the two archaeal *ah* genes included in this analysis (*Haloterrigena daqingensis*) appeared to cluster with the *ah* genes of *Synechococcus* PCC7502, *Calothrix* PCC7507 and *Pseudanabaena* PCC7367 thereby suggesting that a relatively ancient horizontal gene transfer event of *ah* may have occurred from cyanobacteria to Archaea (Supplementary Figure S2). Also, interestingly, the *ah* gene of *Ferrovibrio sp*. and *Magnetospirillium marisnigri* representative of the Rhodospirillaceae family of non-sulfur purple bacteria, appeared to cluster with the deep-rooting cyanobacteria. This finding suggests that the allophanate hydrolase enzyme was present in an “ancient” photosynthetic ancestor, and it was then frequently lost in cyanobacteria. Thirty-six cyanobacteria lack the complete set of urea transport (*urtABCDE*) and catabolism (*ureABCDEFG*, *uc*, and *ah*) genes (Supplementary Table S14), in agreement with the fact that they possess a small genome.

So far, little is known concerning the regulation of the urea transport and catabolism genes. Several studies performed with different cyanobacteria showed that the urease genes are not regulated by changes in nitrogen availabilities or exposure to various stress (see http://cyanoexpress.sysbiolab.eu/). In *Synechocystis* PCC6803, the *urt* genes appears to be strongly regulated (negatively) in response to H_2_O_2_, a high concentration of iron and cadmium, whereas the expression of *ureC* encoding the urease catalytic subunit is almost unchanged (Houot et al., 2007). In *Synechococcus* PCC 7002, the expression of the urease genes were not affected by changes in nitrogen source or nitrogen starvation (Ludwig and Bryant, 2012). In *Microcystis aeruginosa* exposed to nitrogen limitation, all urt genes were significantly upregulated, whereas *ure* transcript levels were not affected (Harke and Gobler, 2013). Furthermore, interestingly, **t**he cyanobacterial PII signal transduction protein was recently shown to operate in the control of the uptake of ammonium, nitrate and urea. First, PII controls ammonium uptake by interacting with the Amt1 ammonium permease. Second, PII mediates the ammonium- and dark-induced inhibition of nitrate uptake by interacting with the NrtC and NrtD subunits of the nitrate/nitrite transporter NrtABCD. PII regulates urea uptake by interacting with the UrtE subunit. The deregulation of urea uptake in a PII deletion mutant causes ammonium excretion when urea is provided as nitrogen source.

## Conclusion

Using a comparative genomic approach, we have analyzed the publicly available genome sequence of 308 cyanobacteria, the photosynthetic prokaryotes that are increasingly studied for basic and applied science. We found that most cyanobacteria harbor all genes encoding the urea transport (*urtABCDE*) and the urea catabolytic enzyme urease (*ureABCDEFG*), in agreement with the capacity of the few tested cyanobacteria to grow on urea as the sole nitrogen source. This finding has major implications for the future engineering of effective cyanobacterial factories for an economically viable production of chemicals coupled to the consumption of urea, which is cheaper than nitrate (the usual nitrogen source) and is frequently present in natural or waste waters. Other cyanobacteria have *ureABCDEFG* or *urtABCDE*, indicating that urease also operates in the detoxification (recycling of carbon and nitrogen) of internally generated urea and that *urtABCDE* could operate in the transport of not only urea but also of other nutrients. Three cyanobacteria of the genera *Gloeobacter* and *Gloeomargarita*, which likely diverged early from other cyanobacteria, have the genes encoding the urea carboxylase (*uc*) and allophanate hydrolase (*ah*) enzymes that sequentially catabolize urea. This finding indicates that the urea carboxylase and allophanate hydrolase enzymes may have appeared in cyanobacteria before urease. The diverse metabolic pathways of urea transport and decomposition of cyanobacteria may contribute to differences in their role in the biogeochemistry of urea, as well as in the role that urea plays in the physiology and ecology of cyanobacteria.

## Author Contributions

FC conceived the project, wrote the manuscript, and agreed to serve as the author responsible for contact and ensures communication. TV and CC-C retrieved all genomic information, conceived the figures and the tables, and read out the manuscript. TV, CC-C, and FC analyzed the data.

## Conflict of Interest Statement

The authors declare that the research was conducted in the absence of any commercial or financial relationships that could be construed as a potential conflict of interest.

## Supplementary Material

FIGURE S1 Organization of the urea acquisition and catabolism genes in various model cyanobacteria.

FIGURE S2 Phylogenetic distribution of the genes encoding the urease (ureC subunit, **A**), allophanate hydrolase **(B)** and urea carboxylase **(C)** proteins in various organisms.

TABLE S1 Distribution of genes involved urea transport and catabolism in 308 cyanobacterial species.

TABLE S2 List of the 264 species of cyanobacteria harboring *ureABC* genes encoding the urease catalytic subunits.

TABLE S3 List of the 255 species of cyanobacteria harboring *ureABC* genes encoding the urease catalytic subunits and the ure*DEFG genes* encoding the urease accessory proteins.

TABLE S4 List of the 40 species of cyanobacteria that possess no *ureABCDEFG* genes in their genome.

TABLE S5 List of the 13 species of cyanobacteria that possess an incomplete set of *ure* genes in their genome.

TABLE S6 List of the 235 species of cyanobacteria harboring *urtABCD* genes involved in the urea transport.

TABLE S7 List of the 225 species of cyanobacteria harboring simultaneously *ureABCDEFG* genes and *urtABCD* genes in their genome.

TABLE S8 List of the 23 species of cyanobacteria harboring *ureABCDEFG* genes without *urtABCDE* genes in their genome.

TABLE S9 List of the 237 species of cyanobacteria harboring a duplication of *ureABCDEFG* genes and/or *urtABCDE* genes in their genome.

TABLE S10 List of the 237 species of cyanobacteria harboring *ureABCDEFG* genes but no urea carboxylase (*uc*) and allophanate hydrolase (*ah*) encoding genes in their genome.

TABLE S11 List of the nine species of cyanobacteria harboring both urea carboxylase (*uc*) and allophanate hydrolase (*ah*) encoding genes in their genome.

TABLE S12 List of the nine species of cyanobacteria harboring both urea carboxylase (*uc*) and allophanate hydrolase (*ah*) encoding genes in their genome.

TABLE S13 Only five species of cyanobacteria harbor all *ureABCDEFG*, *urtABCD uc* and *ah*) encoding genes in their genome.

TABLE S14 List of 36 species of cyanobacteria with no *ure* and urt genes nor any *uc* and *ah* genes in their genome.

TABLE S15 Distribution of *ureABCDEFG*, *urtABCDE, uc* and *ah* genes in model cyanobacteria.

TABLE S16 Properties of model cyanobacteria with a sequenced genome.
