# Supplementary material for: Genomics of Urea Transport and Catabolism in Cyanobacteria: Biotechnological Implications
Source: Front Microbiol. 2019 Sep 4;10:2052. doi: 10.3389/fmicb.2019.02052 (PMC6737895; doi:10.3389/fmicb.2019.02052)
Supplement: FIGURE S1 — Organization of the urea acquisition and catabolism genes in various model cyanobacteria. [file Presentation_1.PPTX]

## Slide 1
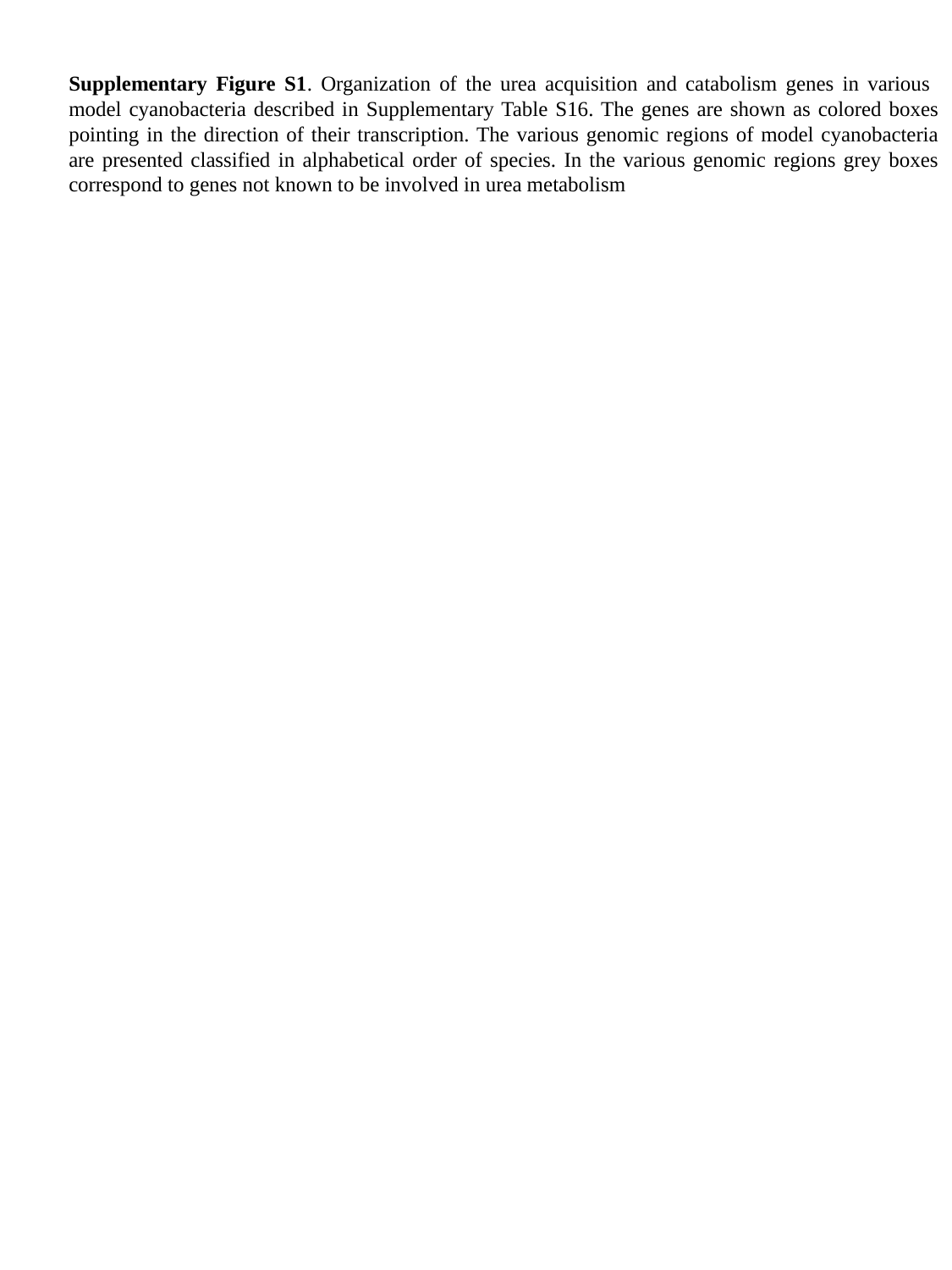

Supplementary Figure S1. Organization of the urea acquisition and catabolism genes in various model cyanobacteria described in Supplementary Table S16. The genes are shown as colored boxes pointing in the direction of their transcription. The various genomic regions of model cyanobacteria are presented classified in alphabetical order of species. In the various genomic regions grey boxes correspond to genes not known to be involved in urea metabolism

## Slide 2
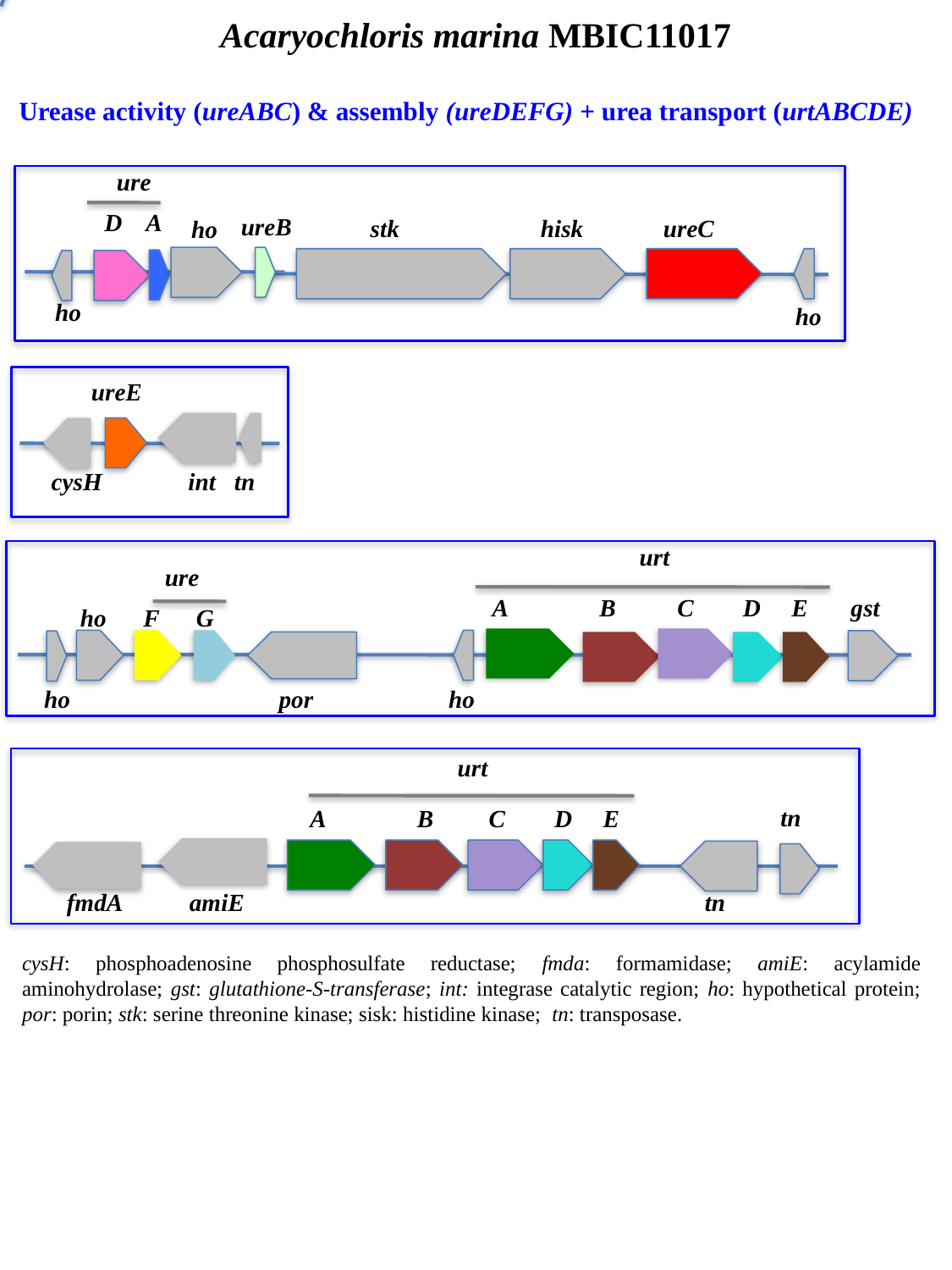

Acaryochloris marina MBIC11017
Urease activity (ureABC) & assembly (ureDEFG) + urea transport (urtABCDE)
 ure
D A
ureB
 stk hisk ureC
 ho
ho
ho
ureE
cysH int tn
 urt
 A B C D E gst
ure
ho F G
 ho por ho
 urt
 A B C D E
fmdA amiE tn
tn
cysH: phosphoadenosine phosphosulfate reductase; fmda: formamidase; amiE: acylamide aminohydrolase; gst: glutathione-S-transferase; int: integrase catalytic region; ho: hypothetical protein; por: porin; stk: serine threonine kinase; sisk: histidine kinase; tn: transposase.

## Slide 3
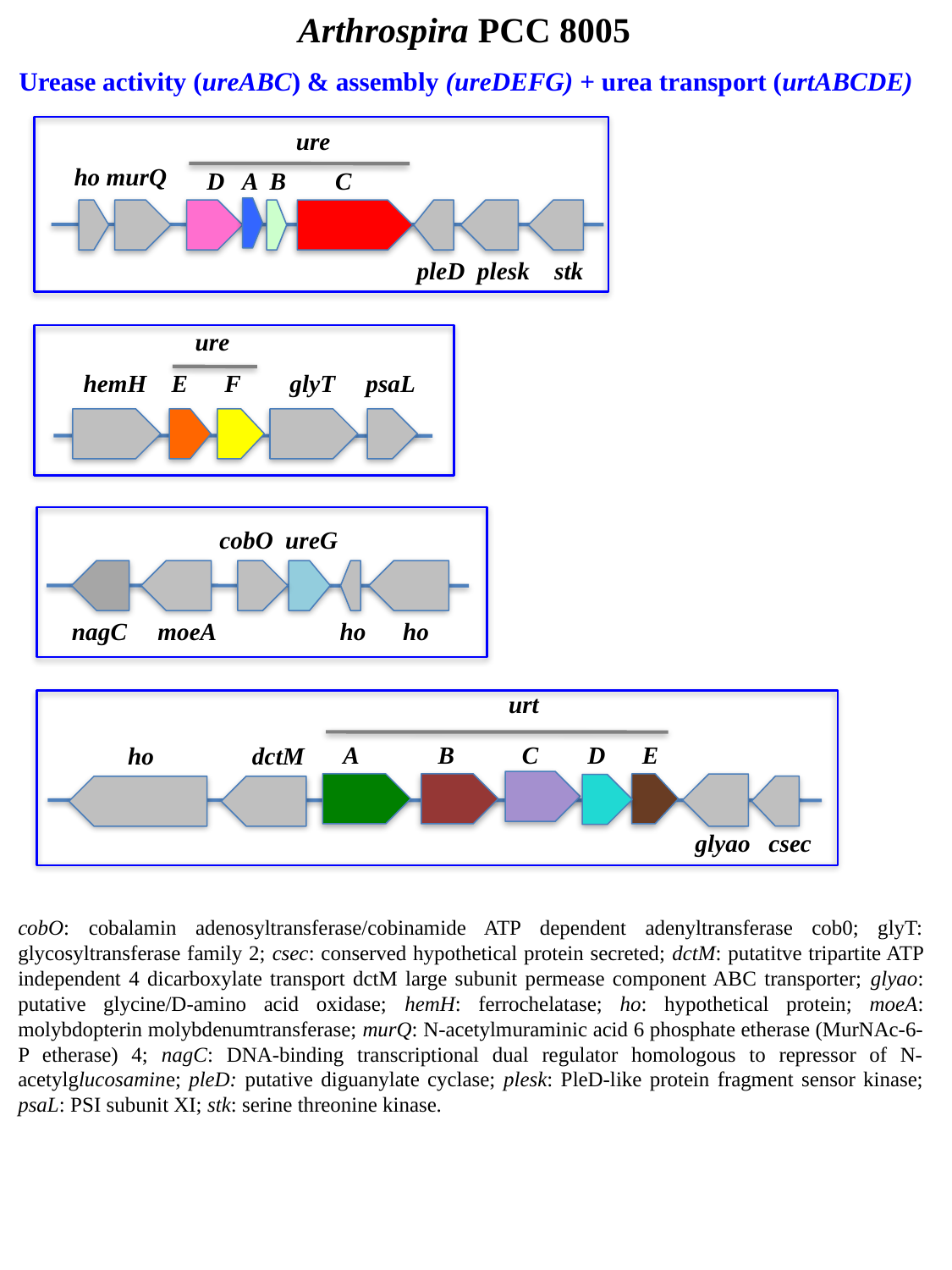

Arthrospira PCC 8005
Urease activity (ureABC) & assembly (ureDEFG) + urea transport (urtABCDE)
ure
D A B C
ho murQ
pleD plesk stk
 ure
hemH E F glyT psaL
 cobO ureG
nagC moeA ho ho
 urt
 A B C D E
 ho dctM
glyao csec
cobO: cobalamin adenosyltransferase/cobinamide ATP dependent adenyltransferase cob0; glyT: glycosyltransferase family 2; csec: conserved hypothetical protein secreted; dctM: putatitve tripartite ATP independent 4 dicarboxylate transport dctM large subunit permease component ABC transporter; glyao: putative glycine/D-amino acid oxidase; hemH: ferrochelatase; ho: hypothetical protein; moeA: molybdopterin molybdenumtransferase; murQ: N-acetylmuraminic acid 6 phosphate etherase (MurNAc-6-P etherase) 4; nagC: DNA-binding transcriptional dual regulator homologous to repressor of N-acetylglucosamine; pleD: putative diguanylate cyclase; plesk: PleD-like protein fragment sensor kinase; psaL: PSI subunit XI; stk: serine threonine kinase.

## Slide 4
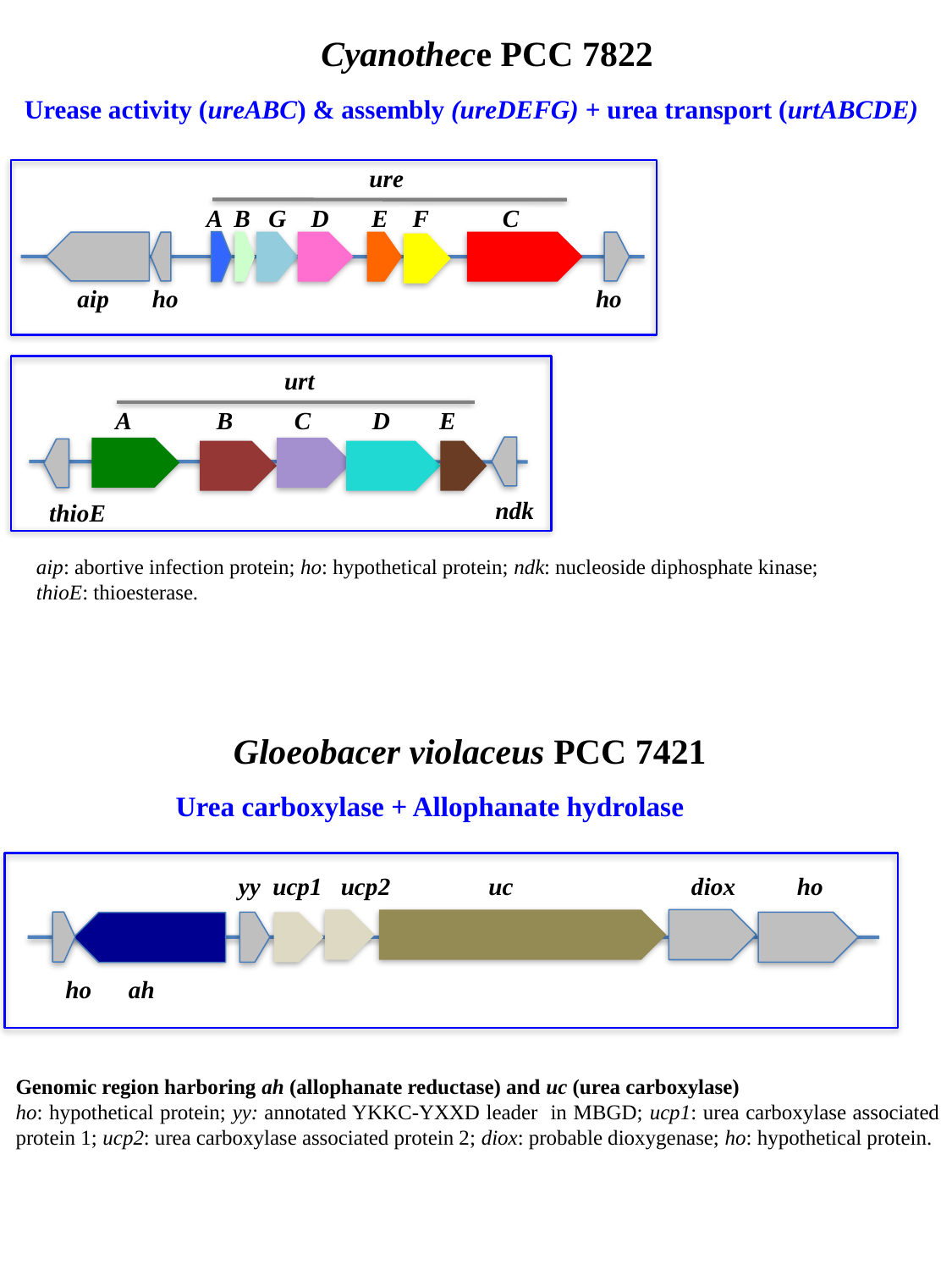

Cyanothece PCC 7822
Urease activity (ureABC) & assembly (ureDEFG) + urea transport (urtABCDE)
ure
A B G D E F C
aip ho ho
 urt
 A B C D E
ndk
thioE
aip: abortive infection protein; ho: hypothetical protein; ndk: nucleoside diphosphate kinase;
thioE: thioesterase.
Gloeobacer violaceus PCC 7421
Urea carboxylase + Allophanate hydrolase
yy ucp1 ucp2 uc diox ho
ho ah
Genomic region harboring ah (allophanate reductase) and uc (urea carboxylase)
ho: hypothetical protein; yy: annotated YKKC-YXXD leader in MBGD; ucp1: urea carboxylase associated protein 1; ucp2: urea carboxylase associated protein 2; diox: probable dioxygenase; ho: hypothetical protein.

## Slide 5
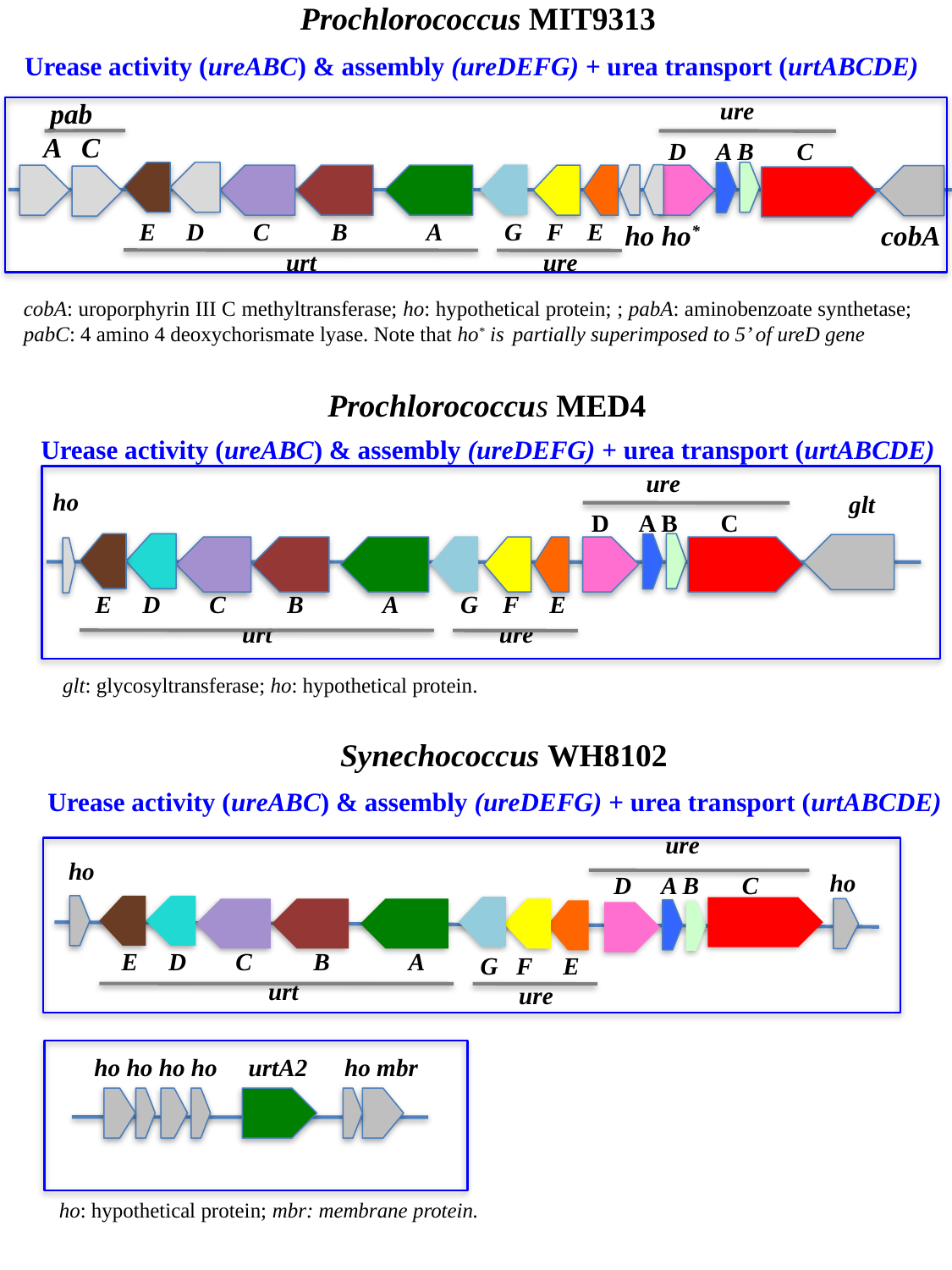

Prochlorococcus MIT9313
Urease activity (ureABC) & assembly (ureDEFG) + urea transport (urtABCDE)
ure
 D A B C
 pab
A C
E D C B A
urt
G F E
ure
ho ho* cobA
cobA: uroporphyrin III C methyltransferase; ho: hypothetical protein; ; pabA: aminobenzoate synthetase; pabC: 4 amino 4 deoxychorismate lyase. Note that ho* is partially superimposed to 5’ of ureD gene
Prochlorococcus MED4
 ure
 D A B C
Urease activity (ureABC) & assembly (ureDEFG) + urea transport (urtABCDE)
glt
ho
E D C B A
urt
G F E
ure
glt: glycosyltransferase; ho: hypothetical protein.
Synechococcus WH8102
Urease activity (ureABC) & assembly (ureDEFG) + urea transport (urtABCDE)
ure
 D A B C
ho
ho
E D C B A
urt
G F E
ure
 ho ho ho ho urtA2 ho mbr
ho: hypothetical protein; mbr: membrane protein.

## Slide 6
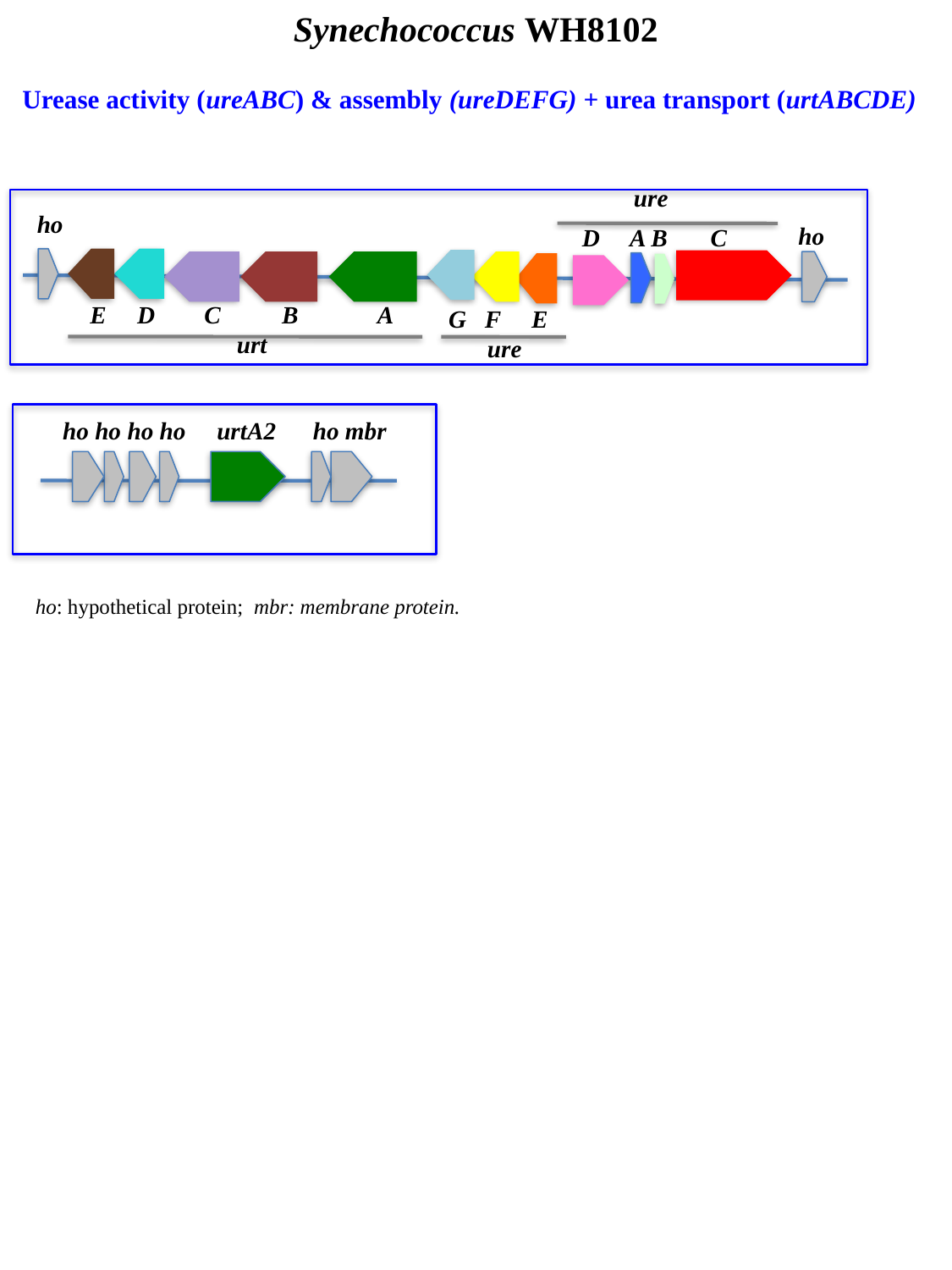

Synechococcus WH8102
Urease activity (ureABC) & assembly (ureDEFG) + urea transport (urtABCDE)
ure
 D A B C
ho
ho
E D C B A
urt
G F E
ure
 ho ho ho ho urtA2 ho mbr
ho: hypothetical protein; mbr: membrane protein.

## Slide 7
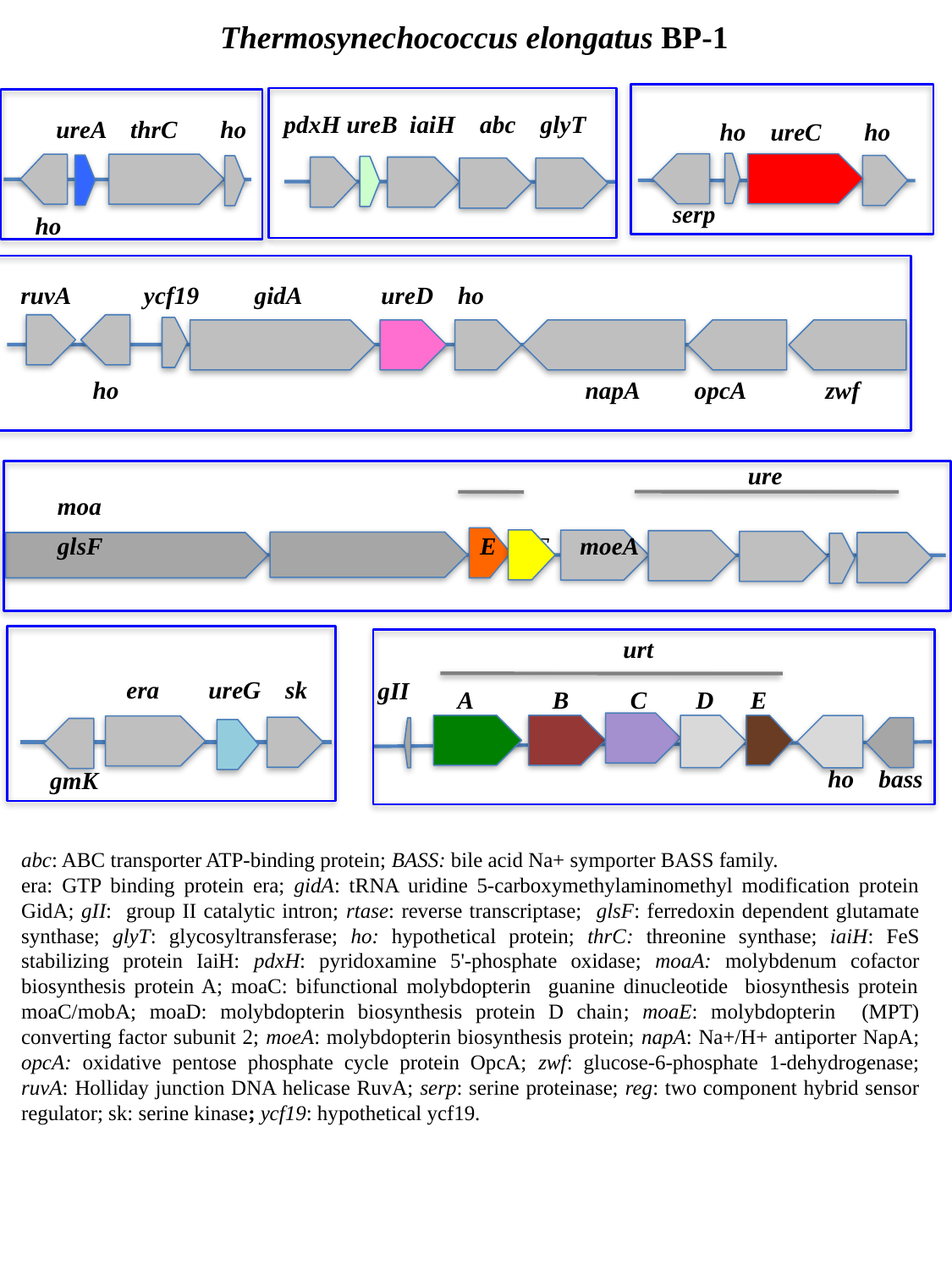

Thermosynechococcus elongatus BP-1
 pdxH ureB iaiH abc glyT
 ureA thrC ho
ho
 ho ureC ho
serp
ruvA ycf19 gidA ureD ho
ho napA opcA zwf
 					 ure moa
glsF reg E F moeA A C D E
 urt
 A B C D E
 gII
ho bass
 era ureG sk
 gmK
abc: ABC transporter ATP-binding protein; BASS: bile acid Na+ symporter BASS family.
era: GTP binding protein era; gidA: tRNA uridine 5-carboxymethylaminomethyl modification protein GidA; gII: group II catalytic intron; rtase: reverse transcriptase; glsF: ferredoxin dependent glutamate synthase; glyT: glycosyltransferase; ho: hypothetical protein; thrC: threonine synthase; iaiH: FeS stabilizing protein IaiH: pdxH: pyridoxamine 5'-phosphate oxidase; moaA: molybdenum cofactor biosynthesis protein A; moaC: bifunctional molybdopterin guanine dinucleotide biosynthesis protein moaC/mobA; moaD: molybdopterin biosynthesis protein D chain; moaE: molybdopterin (MPT) converting factor subunit 2; moeA: molybdopterin biosynthesis protein; napA: Na+/H+ antiporter NapA; opcA: oxidative pentose phosphate cycle protein OpcA; zwf: glucose-6-phosphate 1-dehydrogenase; ruvA: Holliday junction DNA helicase RuvA; serp: serine proteinase; reg: two component hybrid sensor regulator; sk: serine kinase; ycf19: hypothetical ycf19.

## Slide 8
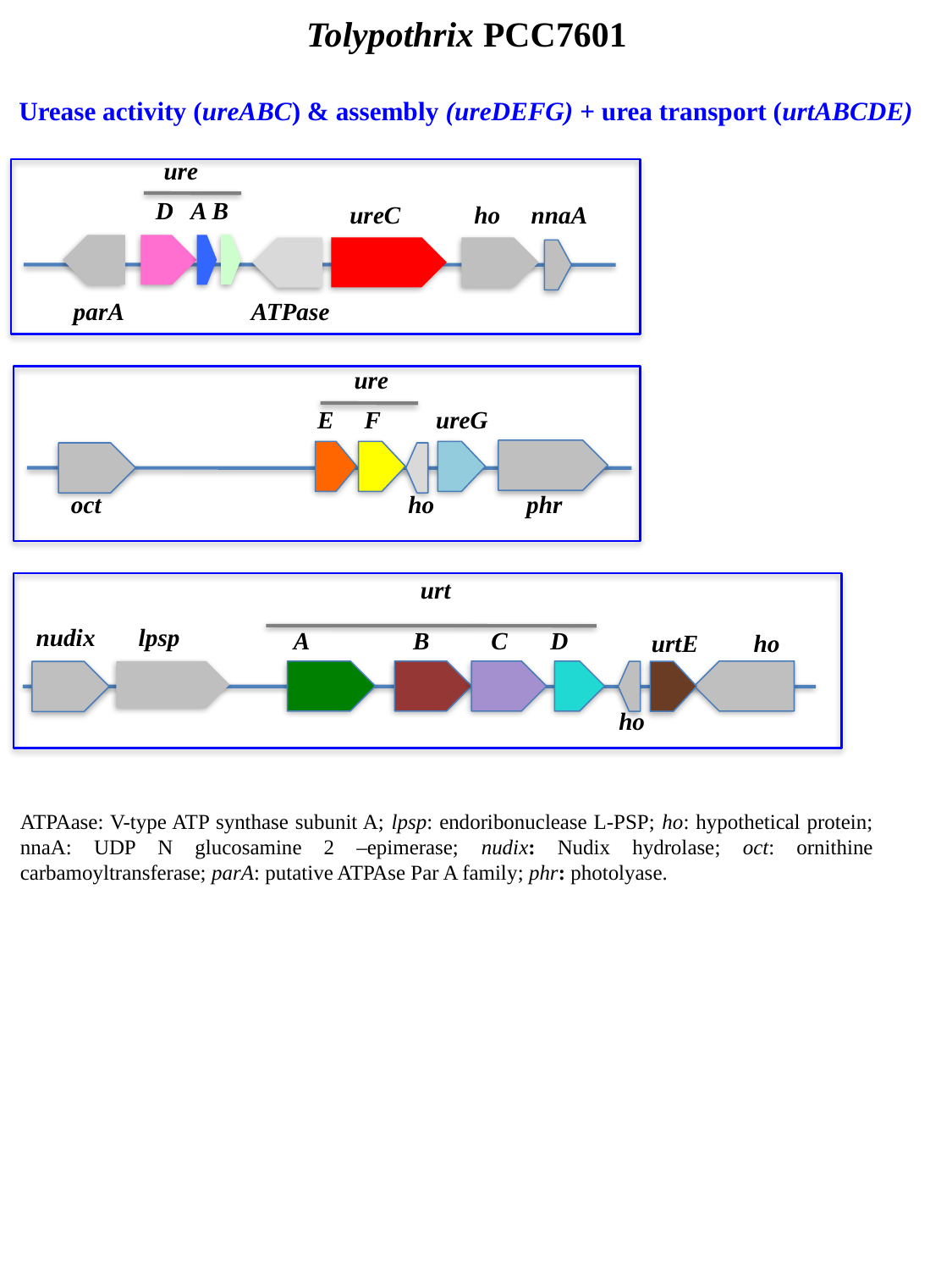

Tolypothrix PCC7601
Urease activity (ureABC) & assembly (ureDEFG) + urea transport (urtABCDE)
 ure
 D A B
ureC ho nnaA
parA ATPase
 ure
E F ureG
oct ho phr
 urt
 A B C D
nudix lpsp
 urtE ho
ho
ATPAase: V-type ATP synthase subunit A; lpsp: endoribonuclease L-PSP; ho: hypothetical protein; nnaA: UDP N glucosamine 2 –epimerase; nudix: Nudix hydrolase; oct: ornithine carbamoyltransferase; parA: putative ATPAse Par A family; phr: photolyase.
