# Supplementary material for: Genomics of Urea Transport and Catabolism in Cyanobacteria: Biotechnological Implications
Source: Front Microbiol. 2019 Sep 4;10:2052. doi: 10.3389/fmicb.2019.02052 (PMC6737895; doi:10.3389/fmicb.2019.02052)
Supplement: FIGURE S2 — Phylogenetic distribution of the genes encoding the urease (ureC subunit, A), allophanate hydrolase (B) and urea carboxylase (C) proteins in various organisms. [file Presentation_2.pptx]

## Slide 1
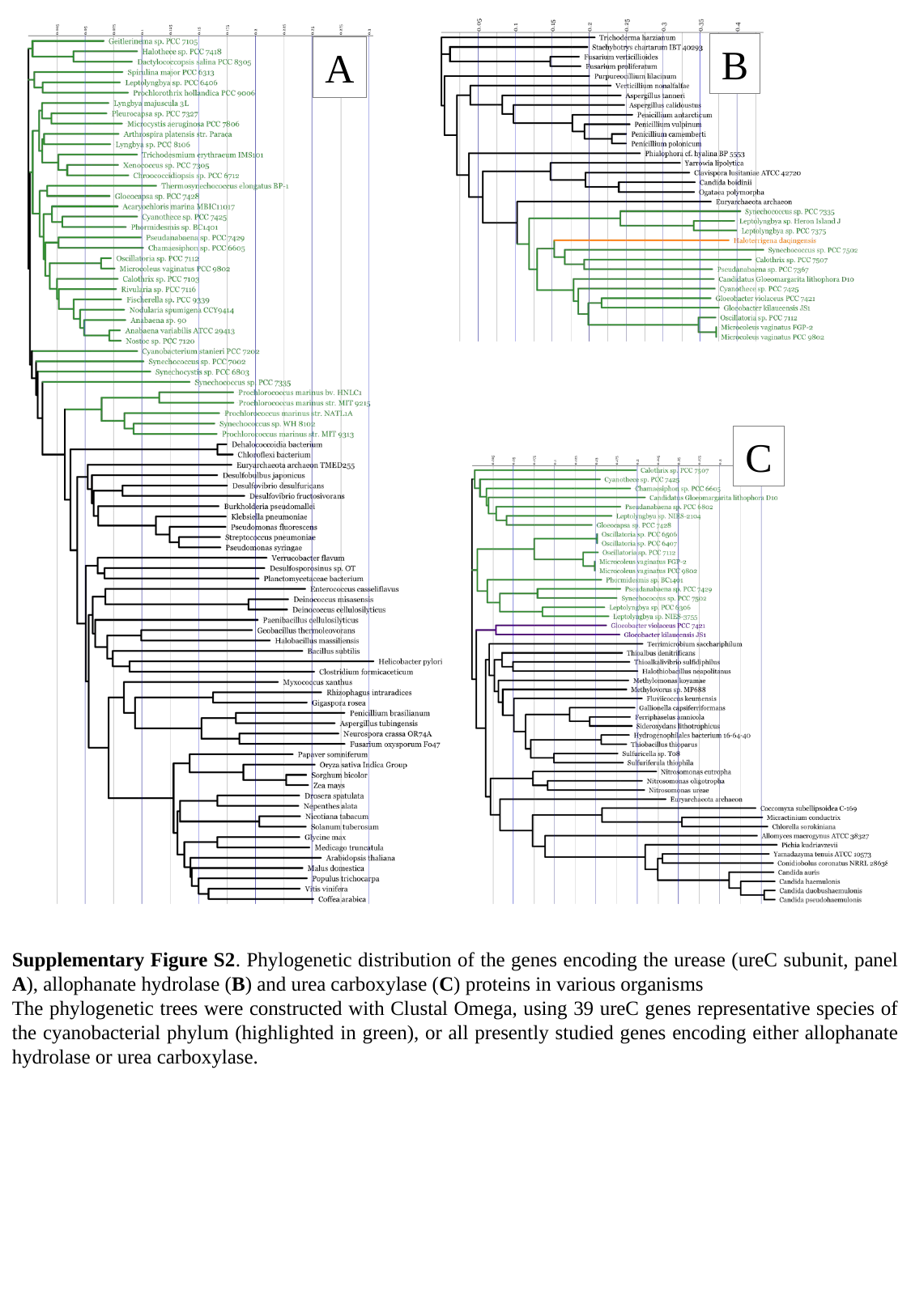

A
B
C
Supplementary Figure S2. Phylogenetic distribution of the genes encoding the urease (ureC subunit, panel A), allophanate hydrolase (B) and urea carboxylase (C) proteins in various organisms
The phylogenetic trees were constructed with Clustal Omega, using 39 ureC genes representative species of the cyanobacterial phylum (highlighted in green), or all presently studied genes encoding either allophanate hydrolase or urea carboxylase.
