# Supplementary material for: Genomics of Urea Transport and Catabolism in Cyanobacteria: Biotechnological Implications
Source: Front Microbiol. 2019 Sep 4;10:2052. doi: 10.3389/fmicb.2019.02052 (PMC6737895; doi:10.3389/fmicb.2019.02052)
Supplement: TABLE S16 — Properties of model cyanobacteria with a sequenced genome. [file Data_Sheet_1.PDF]

Supplementary Table S16. Properties of model cyanobacteria with a sequenced genome

Name of cyanobacteria	Genome size & complexity	Morphology	Properties of the strain	N° of articles
Acaryochloris marina MBIC11017	**8.36 Mb**<br>1 circular chromosome<br>9 plasmids<br>(Swingley *et al.*, 2008)	Unicellular	- Synthetizes **Chlorophyll *d*** instead of chlorophyll *a* and can grow under far-red light (Hernández-Prieto *et al.*, 2018)<br>- PS & CO_2 assimilation genes are located on plasmids<br>- 2 *sodA*, 1 *sodC*, 1 *sodN*, 1 *katG*; 1 *gr*; 7 *recA* (4 located on a different plasmid).<br>- 1 *gap1*, 1 *gap2*<br>- Possesses Urea acquisition and catabolism genes (*ure* and *urt* genes)	143
Arthrospira PCC8005	**6.28 Mb**<br>1 circular chromosome<br>0 plasmid<br>(Xu *et al.*, 2016)	Filamentous	- Typically resides in alkaline lakes<br>- **Edible,** rich in vitamin B12 and fatty acid with a long history of human consumption (Janssen *et al.*, 2010)<br>- Radiation resistant (Badri *et al.*, 2015)<br>- Selected by the European Space Agency as an O_2 producer and a nutritional end product of the MELiSSA life support system<br>- 1 *sodB*, 0 *cat,* 1*gr*<br>-1 *gap1*, *gap2*<br>- Better growth in presence of urea than nitrate (Deschoenmaeker *et al.*, 2017)<br>The activity of urease is increased in N-deprivation conditions<br>- Possesses all urea transport and uease encoding genes (*ure* and *urt* genes)	12
Anabaena PCC7120<br><br>or *Nostoc* PCC7120	**7.21 Mb**<br>1 circular chromosome<br>6 plasmids<br>(Kaneko *et al.*, 2001)	Filamentous<br>Heterocystous	- **Best studied filamentous N_2-fixing** cyanobacterium<br>- **Powerful genetics**; well studied for biotechnological purposes (Sun et al., 2018)<br>- *1 sodA* (Ke *et al.*, 2014; Zhao *et al.*, 2007), 1 *sodB* (Regelsberger *et al.*, 2004), 1 *katG*, 1 *gr*<br>- 1*gap1*, 1*gap2*, 1*gap3*<br>- Possesses all urea transport and urease encoding genes (*ure* and *urt* genes)	838
Cyanothece ATCC51142	**5.46 Mb**<br>1 circular chromosome<br>1 linear chromosome<br>4 plasmids<br>(Welsh *et al.*, 2008)	Unicellular	- **Diazotrophic**: Photosynthesis during the day and N_2 fixation at night.<br>- **Grow faster on glycerol**-containing medium<br>- 1 *sodB* (Priya *et al.*, 2007), 0 cat, 1 *gr*, 4 *ruvC*<br>- *gap*1, *gap2*<br>- No urea acquisition and catabolism genes	80
Cyanothece CCY0110	**5.88 Mb**<br>1 circular chromosome<br>0 plasmid<br>http://genome.microbedb.jp/cyanobase/GCA_000169335.1	Unicellular	- **Diazotrophic** : Photosynthesis during the day and N_2 fixation at night<br>- Efficient producer of exopolysaccharides<br>- 1 *sodB* (Priya *et al.*, 2007), **1 Mn-cat** , 1 *katG* (cat/peroxidase), 1 *gr*<br>- *gap1*, *gap2*<br>- No urea acquisition and catabolism genes	5

Name of cyanobacteria	Genome size & complexity	Morphology	Properties of the strain	N° of articles
Cyanothece PCC7425	**5.79 Mb**<br>1 circular chromosome<br>3 plasmids<br>(Bandyopadhyay *et al.*, 2011)	Unicellular	- **Anaerobic N_2-fixation.**<br>- 1 *sodA,* 1 *sodB* (Kesheri et al., 2014), 1 *Mn-cat ,* 1*cat;* 1 *gr*<br>- 2 *gap1*, 1 *gap2*<br>- **Complete panoply of urea acquisition and catabolism genes** (*ure* and *urt* genes and *uc* & *ah*)	10
Cyanothece PCC7822	**7.84 Mb**<br>1 circular chromosome<br>3 linear chromosomes<br>3 plasmids<br>(Bandyopadhyay *et al.*, 2011)	Unicellular	- **Large cells (6-10 µm)**<br>- **Diazotrophic:** PS during day & N_2 fixation at night).<br>- Synthetizes **phycoerythrin**<br>- can produce **bioplastics** (polyhydroxyalkanoate)<br>- 1 *sodA,* 1 *sodB*, *2 cat*; 1 *gr*, 4 *ruvC*<br>- 1 *gap1*, *gap2*<br>- Urea acquisition and catabolism genes (*ure* and *urt* genes)	11
Fremyella diplosiphon PCC7601<br><br>Also named:<br>*(Tolypothrix)* PCC7601,<br>Calothrix	**9.97 Mb**<br>(Yerrapragada *et al.*, 2015)	Filamentous Heterocystous	- **N_2-fixing, heterocystous** cyanobacterium<br>- Differentiates **hormogonia**<br>- Synthetizes **phycoerythrin**<br>- Performs **complementary chromatic adaptation**: Modification of cell morphology and photosynthetic pigment content according to light (quality & intensity) (Singh and Montgomery, 2015)<br>- Amenable to gene manipulation (Singh and Montgomery, 2015)<br>- 1 *sodA*, 1 *sodB*, 1 *katG* , 1*cathpi*, 2 *gr*<br>- 1 *gap1*, 1 *gap2*, 1 *gap3*<br>- Urea acquisition and catabolism genes (*ure* and *urt* genes)	113
Gloeobacter violaceus PCC7421	**4.66 Mb**<br>1 circular chromosome<br>0 plasmid<br>(Nakamura *et al.*, 2003)	Unicellular	- Synthetizes **phycoerythrin**<br>- No thylakoids<br>- Obligatory photoautotrophic<br>- Produces hopanoids<br>- **Likely diverged from all cyanobacteria before the evolution of thylakoids.**<br>- 1 *sodA*, 1 *sodB,* 2 *sodC* (Priya *et al.*, 2007), 2 *cat*, 1 *gr*<br>- 1 *gap1*, 1 *gap2*<br>- No urea acquisition and catabolism ure and urt genes. Possesses uc and ah genes the involved in urea catabolismc *uc* & *ah*)	153
Prochlorococcus marinus SS120<br>Also named CCMP1375	**1.75 Mb**<br>1 circular chromosome<br>0 plasmids<br>(Dufresne *et al.*, 2003)	Unicellular	- **Globally-abundant marine** cyanobacterium; adapted to low-light<br>- Synthetizes **phycoerythrin** and **No phycocyanin**<br>- **Can use exogenous glucose** as an extra resource of carbon and energy.<br>- 1 *sodN*, 0 *cat*, 1 *gr*<br>- 1 *gap1*, 1 *gap2*<br>- No urea acquisition and catabolism genes	23

Name of cyanobacteria	Genome size & complexity	Morphology	Properties of the strain	N° of articles
Prochlorococcus marinus MIT9313	**2.41 Mb**<br>1 circular chromosome<br>0 plasmid<br>(Rocap *et al.*, 2003)	Unicellular	- **Globally-abundant marine** cyanobacterium; adapted to low-light<br>**- No phycocyanin**<br>- **Can grow on urea** as nitrogen source and **glucose** as carbon source<br>- 1 *sodN* (Priya *et al.*, 2007); **0 *cat***; 1 *gr*<br>- 1 *gap1*, 1 *gap2*.<br>- Urea acquisition and catabolism genes (*ure* and *urt* genes)	15
Prochlorococcus marinus MED4<br>Also named<br>subsp. Pastoris CCMP1986	**1.66 Mb**<br>1 circular chromosome<br>0 plasmid<br>(Rocap *et al.*, 2003)	Unicellular	- **Globally-abundant marine** cyanobacterium; adapted to high-light<br>**- Does not synthetize phycocyanin and allophycocyanin**<br>- **Can grow on urea** as nitrogen source and **glucose** as carbon source.<br>- 1 *sodN* (Priya *et al.*, 2007); **0 *cat;*** 1 *gr*<br>- Urea acquisition and catabolism genes (*ure* and *urt* genes)	27
Synechococcus elongatus PCC7942<br>formerly named<br>*"Anacystis nidulans* R2*"*	**2.74 Mb**<br>1 circular chromosome<br>2 plasmids<br>(Herbert *et al.*, 1992)	Unicellular	- Fresh-water.<br>- Obligate photoautotroph<br>- **Circadian rhythm**<br>- **Powerful genetics** (Taton *et al.*, 2014)<br>**- Well studied for biotechnological** purposes (Sun *et al.*, 2018).<br>- 1 *cat*; 1 *gr,* 1 *sodB* (Herbert *et al.*, 1992)(Thomas *et al.*, 1998)<br>- 1 *gap1*, 1 *gap2, 1 gap3*<br>- No urea acquisition and catabolism genes	981
Synechococcus PCC7002<br><br>Formerly named<br>"*Agmenellum quadruplicatum* PR-6"	**3.41 Mb**<br>1 circular chromosome<br>7 plasmids<br>(Ruffing *et al.*, 2016)	Unicellular	- **Marine;** Euryhaline;<br>- **Tolerant to very high light**<br>- **Requires vitamin B12; Fast growth, accelerated by glycerol**<br>- Produce polyhydroxyalkanoate<br>- **Powerful genetics**; Well studied for biotechnological purposes (Sun *et al.*, 2018)<br>- 1*gap1*, 1 *gap2*<br>- 1 *sodB*, 1 *katG*, 1 *gr*<br>- **Can grow on urea** as nitrogen source<br>- Urea acquisition and catabolism genes (*ure* and *urt* genes)	369
Synechococcus WH7803<br><br>Also named RCC752<br>DC2, CCMP1334	**2.37 Mb**<br>1 circular chromosome<br>0 plasmid<br>(Six *et al.*, 2007)	Unicellular	- **Globally-abundant marine** cyanobacterium<br>- Synthetizes **phycoerythrin**<br>- Amenable to genetic tranformation<br>- 1 *katG*, 1 gr, 1*sodB,* 1 *sodC* (Chadd *et al.*, 1996)<br>- **No gene encoding Ni-containing enzymes** (urease, hydrogenase & SodA)	52
Synechococcus WH8102<br><br>Also named RCC539<br>CCMP2370	**2.43 Mb**<br>1 circular chromosome<br>0 plasmid<br>(Palenik *et al.*, 2003)	Unicellular	- **Globally-abundant marine** cyanobacterium<br>- Motile<br>- Amenable to genetic transformation<br>- 1*sodN* (Priya *et al.*, 2007; Qiu and Price, 2009), **0 *cat***<br>- Urea acquisition and catabolism genes (*ure* and *urt* genes)	62

Name of cyanobacteria	Genome size & complexity	Morphology	Properties of the strain	N° of articles
Synechocystis PCC6803<br>*Synechocystis* ATCC 27184	**3.95 Mb**<br>1 circular chromosome<br>7 plasmids<br>(Kaneko *et al.*, 1996)	Unicellular	- Euryhaline, Motile<br>- Facultative heterotroph (glucose). Glycerol is toxic<br>- Produce polyhydroxyalkanoate and abundant & complex **exopolysaccharides**<br>- **Powerful genetics**; Well studied for biotechnological purposes (Sun *et al.*, 2018)<br>- *1 sodB* (Priya *et al.*, 2007), 1 *katG*, 0 *gr*<br>- 1 *gap1*, 1 *gap2*<br>- **Can grow on urea**<br>- Urea acquisition and catabolism genes (*ure* and *urt* genes)	4109
Thermosynechococcus elongatus BP-1	**2.59 Mb**<br>1 circular chromosome<br>0 plasmid	Unicellular	**- Thermophilic**<br>**- Well studied for photosynthesis.**<br>- *1 sodA* (Priya *et al.*, 2007), 1 *sodB* (Priya *et al.*, 2007), 0 *cat*, 1 *gr*	**88**

Abbreviations:
cat: encodes catalase
Chl: chlorophyll,
gap: glyceraldehyde-3P-dehydrogenase encoding genes: *gap1*, *gap2*, *gap3*.
gr: encodes glutathione reductase
katG; encodes catalase peroxidase
PHA: polyhydroxyalkanoates
PS: photosynthesis
SOD: superoxide dismutase
sodA :encodes a Mn/Fe-SOD
sodB : encodes a Fe-SOD
sodC: encodes a Cu-ZnSOD
sodN: encodes a Ni-SOD

Number of articles found in the Pubmed database by May15th, 2019

References cited in Supplementary Table S16

Badri, H., Monsieurs, P., Coninx, I., Wattiez, R., and Leys, N. (2015) Molecular investigation of the radiation resistance of edible cyanobacterium Arthrospira sp. PCC 8005. *MicrobiologyOpen* **4**: 187–207.

Bandyopadhyay, A., Elvitigala, T., Welsh, E., Stöckel, J., Liberton, M., Min, H., et al. (2011) Novel metabolic attributes of the genus Cyanothece, comprising a group of unicellular nitrogen-fixing cyanobacteria. *mBio* **2**:.

Chadd, H.E., Newman, J., Mann, N.H., and Carr, N.G. (1996) Identification of iron superoxide dismutase and a copper/zinc superoxide dismutase enzyme activity within the marine cyanobacterium Synechococcus sp. WH 7803. *FEMS Microbiology Letters* **138**: 161–5.

Deschoenmaeker, F., Bayon-Vicente, G., Sachdeva, N., Depraetere, O., Cabrera Pino, J.C., Leroy, B., et al. (2017) Impact of different nitrogen sources on the growth of Arthrospira sp. PCC 8005 under batch and continuous cultivation – A biochemical, transcriptomic and proteomic profile. *Bioresource Technology* **237**: 78–88.

Dufresne, A., Salanoubat, M., Partensky, F., Artiguenave, F., Axmann, I.M., Barbe, V., et al. (2003) Genome sequence of the cyanobacterium Prochlorococcus marinus SS120, a nearly minimal oxyphototrophic genome. *Proceedings of the National Academy of Sciences* **100**: 10020–5.

Herbert, S.K., Samson, G., Fork, D.C., and Laudenbach, D.E. (1992) Characterization of damage to photosystems I and II in a cyanobacterium lacking detectable iron superoxide dismutase activity. *Proc. Natl. Acad. Sci. USA* **89**: 8716–20.

Hernández-Prieto, M.A., Li, Y., Postier, B.L., Blankenship, R.E., and Chen, M. (2018) Far-red light promotes biofilm formation in the cyanobacterium Acaryochloris marina. *Environmental Microbiology* **20**: 535–545.

Janssen, P.J., Morin, N., Mergeay, M., Leroy, B., Wattiez, R., Vallaeys, T., et al. (2010) Genome sequence of the edible cyanobacterium Arthrospira sp. PCC 8005. *Journal of Bacteriology* **192**: 2465–6.

Kaneko, T., Nakamura, Y., Wolk, C.P., Kuritz, T., Sasamoto, S., Watanabe, A., et al. (2001) Complete genomic sequence of the filamentous nitrogen-fixing cyanobacterium Anabaena sp. strain PCC 7120. *DNA research : an international journal for rapid publication of reports on genes and genomes* **8**: 227–53.

Kaneko, T., Sato, S., Kotani, H., Tanaka, A., Asamizu, E., Nakamura, Y., et al. (1996) Sequence analysis of the genome of the unicellular cyanobacterium synechocystis sp. strain PCC6803. II. Sequence determination of the entire genome and assignment of potential protein-coding regions. *DNA Research* **3**: 109–36.

Ke, W.T., Dai, G.Z., Jiang, H.B., Zhang, R., and Qiu, B.S. (2014) Essential roles of iron superoxide dismutase in photoautotrophic growth of Synechocystis sp. PCC 6803 and heterogeneous expression of marine Synechococcus sp. CC9311 copper/zinc superoxide dismutase within its sodB knockdown mutant. *Microbiology (United Kingdom)* **160**: 228–41.

Kesheri, M., Kanchan, S., Richa, and Sinha, R.P. (2014) Isolation and in silico analysis of Fe-superoxide dismutase in the cyanobacterium Nostoc commune. *Gene* **553**: 117–25.

Nakamura, Y., Kaneko, T., Sato, S., Ikeuchi, M., Katoh, H., Sasamoto, S., et al. (2002) Complete genome structure of the thermophilic cyanobacterium Thermosynechococcus elongatus BP-1. *DNA Research* **9**: 123–30.

Nakamura, Y., Kaneko, T., Sato, S., Mimuro, M., Miyashita, H., Tsuchiya, T., et al. (2003) Complete Genome Structure of Gloeobacter violaceus PCC 7421, a Cyanobacterium that Lacks Thylakoids. *DNA Research* **10**: 137–45.

Palenik, B., Brahamsha, B., Larimer, F.W., Land, M., Hauser, L., Chain, P., et al. (2003) The genome of a motile marine Synechococcus. *Nature* **424**:.

Priya, B., Premanandh, J., Dhanalakshmi, R.T., Seethalakshmi, T., Uma, L., Prabaharan, D., and Subramanian, G. (2007) Comparative analysis of cyanobacterial superoxide dismutases to discriminate canonical forms. *BMC Genomics* **8**: 435–45.

Qiu, B. and Price, N.M. (2009) Different physiological responses of four marine synechococcus strains (cyanophyceae) to nickel starvation under iron-replete and iron-deplete conditions. *Journal of Phycology* **45**: 1062–71.

Rocap, G., Larimer, F.W., Lamerdin, J., Malfatti, S., Chain, P., Ahlgren, N.A., et al. (2003) Genome divergence in two Prochlorococcus ecotypes reflects oceanic niche differentiation. *Nature* **424**: 1042–7.

Ruffing, A.M., Jensen, T.J., and Strickland, L.M. (2016) Genetic tools for advancement of Synechococcus sp. PCC 7002 as a cyanobacterial chassis. *Microbial Cell Factories* **15**:.

Singh, S.P. and Montgomery, B.L. (2015) Regulation of BolA abundance mediates morphogenesis in Fremyella diplosiphon. *Frontiers in Microbiology* **5**:.

Six, C., Thomas, J.C., Garczarek, L., Ostrowski, M., Dufresne, A., Blot, N., et al. (2007) Diversity and evolution of phycobilisomes in marine Synechococcus spp.: A comparative genomics study. *Genome Biology* **8**:.

Sun, T., Li, S., Song, X., Diao, J., Chen, L., and Zhang, W. (2018) Toolboxes for cyanobacteria: Recent advances and future direction. *Biotechnology Advances* **36**: 1293–1307.

Swingley, W.D., Chen, M., Cheung, P.C., Conrad, A.L., Dejesa, L.C., Hao, J., et al. (2008) Niche adaptation and genome expansion in the chlorophyll d-producing cyanobacterium Acaryochloris marina. *Proceedings of the National Academy of Sciences* **105**: 2005–10.

Taton, A., Unglaub, F., Wright, N.E., Zeng, W.Y., Paz-Yepes, J., Brahamsha, B., et al. (2014) Broad-host-range vector system for synthetic biology and biotechnology in cyanobacteria. *Nucleic Acids Research* **42**:.

Thomas, D.J., Avenson, T.J., Thomas, J.B., and Herbert, S.K. (1998) A Cyanobacterium Lacking Iron Superoxide Dismutase Is Sensitized to Oxidative Stress Induced with Methyl Viologen but Is Not Sensitized to Oxidative Stress Induced with Norflurazon1. *Plant physiology* **116**: 1593–1602.

Welsh, E.A., Liberton, M., Stöckel, J., Loh, T., Elvitigala, T., Wang, C., et al. (2008) The genome of Cyanothece 51142, a unicellular diazotrophic cyanobacterium important in the marine nitrogen cycle. *Proceedings of the National Academy of Sciences of the USA* **105**: 15094–9.

Xu, T., Qin, S., Hu, Y., Song, Z., Ying, J., Li, P., et al. (2016) Whole genomic DNA sequencing and comparative genomic analysis of Arthrospira platensis: High genome plasticity and genetic diversity. *DNA Research* **23**: 325–338.

Yerrapragada, S., Shukla, A., Hallsworth-Pepin, K., Choi, K., Wollam, A., Clifton, S., et al. (2015) Extreme Sensory Complexity Encoded in the 10-Megabase Draft Genome Sequence of the Chromatically Acclimating Cyanobacterium Tolypothrix sp. PCC 7601. *Genome Announcements*.

Zhao, W., Guo, Q., and Zhao, J. (2007) A membrane-associated Mn-superoxide dismutase protects the photosynthetic apparatus and nitrogenase from oxidative damage in the cyanobacterium Anabaena sp. PCC 7120. *Plant and Cell Physiology* **48**: 563–72.

Badri, H., Monsieurs, P., Coninx, I., Wattiez, R., and Leys, N. (2015) Molecular investigation of the radiation resistance of edible cyanobacterium Arthrospira sp. PCC 8005. *MicrobiologyOpen* **4**: 187–207.

Bandyopadhyay, A., Elvitigala, T., Welsh, E., Stöckel, J., Liberton, M., Min, H., et al. (2011) Novel metabolic attributes of the genus Cyanothece, comprising a group of unicellular nitrogen-fixing cyanobacteria. *mBio* **2**:.

Chadd, H.E., Newman, J., Mann, N.H., and Carr, N.G. (1996) Identification of iron superoxide dismutase and a copper/zinc superoxide dismutase enzyme activity within the marine cyanobacterium Synechococcus sp. WH 7803. *FEMS Microbiology Letters* **138**: 161–5.

Deschoenmaeker, F., Bayon-Vicente, G., Sachdeva, N., Depraetere, O., Cabrera Pino, J.C., Leroy, B., et al. (2017) Impact of different nitrogen sources on the growth of Arthrospira sp. PCC 8005 under batch and continuous cultivation – A biochemical, transcriptomic and proteomic profile. *Bioresource Technology* **237**: 78–88.

Dufresne, A., Salanoubat, M., Partensky, F., Artiguenave, F., Axmann, I.M., Barbe, V., et al. (2003) Genome sequence of the cyanobacterium Prochlorococcus marinus SS120, a nearly minimal oxyphototrophic genome. *Proceedings of the National Academy of Sciences* **100**: 10020–5.

Herbert, S.K., Samson, G., Fork, D.C., and Laudenbach, D.E. (1992) Characterization of damage to photosystems I and II in a cyanobacterium lacking detectable iron superoxide dismutase activity. *Proc. Natl. Acad. Sci. USA* **89**: 8716–20.

Hernández-Prieto, M.A., Li, Y., Postier, B.L., Blankenship, R.E., and Chen, M. (2018) Far-red light promotes biofilm formation in the cyanobacterium Acaryochloris marina. *Environmental Microbiology* **20**: 535–545.

Janssen, P.J., Morin, N., Mergeay, M., Leroy, B., Wattiez, R., Vallaeys, T., et al. (2010) Genome sequence of the edible cyanobacterium Arthrospira sp. PCC 8005. *Journal of Bacteriology* **192**: 2465–6.

Kaneko, T., Nakamura, Y., Wolk, C.P., Kuritz, T., Sasamoto, S., Watanabe, A., et al. (2001) Complete genomic sequence of the filamentous nitrogen-fixing cyanobacterium Anabaena sp. strain PCC 7120. *DNA research : an international journal for rapid publication of reports on genes and genomes* **8**: 227–53.

Kaneko, T., Sato, S., Kotani, H., Tanaka, A., Asamizu, E., Nakamura, Y., et al. (1996) Sequence analysis of the genome of the unicellular cyanobacterium synechocystis sp. strain PCC6803. II. Sequence determination of the entire genome and assignment of potential protein-coding regions. *DNA Research* **3**: 109–36.

Ke, W.T., Dai, G.Z., Jiang, H.B., Zhang, R., and Qiu, B.S. (2014) Essential roles of iron superoxide dismutase in photoautotrophic growth of Synechocystis sp. PCC 6803 and heterogeneous expression of marine Synechococcus sp. CC9311 copper/zinc superoxide dismutase within its sodB knockdown mutant. *Microbiology (United Kingdom)* **160**: 228–41.

Kesheri, M., Kanchan, S., Richa, and Sinha, R.P. (2014) Isolation and in silico analysis of Fe-superoxide dismutase in the cyanobacterium Nostoc commune. *Gene* **553**: 117–25.

Nakamura, Y., Kaneko, T., Sato, S., Ikeuchi, M., Katoh, H., Sasamoto, S., et al. (2002) Complete genome structure of the thermophilic cyanobacterium Thermosynechococcus elongatus BP-1. *DNA Research* **9**: 123–30.

Nakamura, Y., Kaneko, T., Sato, S., Mimuro, M., Miyashita, H., Tsuchiya, T., et al. (2003) Complete Genome Structure of Gloeobacter violaceus PCC 7421, a Cyanobacterium that Lacks Thylakoids. *DNA Research* **10**: 137–45.

Palenik, B., Brahamsha, B., Larimer, F.W., Land, M., Hauser, L., Chain, P., et al. (2003) The genome of a motile marine Synechococcus. *Nature* **424**:.

Priya, B., Premanandh, J., Dhanalakshmi, R.T., Seethalakshmi, T., Uma, L., Prabaharan, D., and Subramanian, G. (2007) Comparative analysis of cyanobacterial superoxide dismutases to discriminate canonical forms. *BMC Genomics* **8**: 435–45.

Qiu, B. and Price, N.M. (2009) Different physiological responses of four marine synechococcus strains (cyanophyceae) to nickel starvation under iron-replete and iron-deplete conditions. *Journal of Phycology* **45**: 1062–71.

Rocap, G., Larimer, F.W., Lamerdin, J., Malfatti, S., Chain, P., Ahlgren, N.A., et al. (2003) Genome divergence in two Prochlorococcus ecotypes reflects oceanic niche differentiation. *Nature* **424**: 1042–7.

Ruffing, A.M., Jensen, T.J., and Strickland, L.M. (2016) Genetic tools for advancement of Synechococcus sp. PCC 7002 as a cyanobacterial chassis. *Microbial Cell Factories* **15**:.

Singh, S.P. and Montgomery, B.L. (2015) Regulation of BolA abundance mediates morphogenesis in Fremyella diplosiphon. *Frontiers in Microbiology* **5**:.

Six, C., Thomas, J.C., Garczarek, L., Ostrowski, M., Dufresne, A., Blot, N., et al. (2007) Diversity and evolution of phycobilisomes in marine Synechococcus spp.: A comparative genomics study. *Genome Biology* **8**:.

Sun, T., Li, S., Song, X., Diao, J., Chen, L., and Zhang, W. (2018) Toolboxes for cyanobacteria: Recent advances and future direction. *Biotechnology Advances* **36**: 1293–1307.

Swingley, W.D., Chen, M., Cheung, P.C., Conrad, A.L., Dejesa, L.C., Hao, J., et al. (2008) Niche adaptation and genome expansion in the chlorophyll d-producing cyanobacterium Acaryochloris marina. *Proceedings of the National Academy of Sciences* **105**: 2005–10.

Taton, A., Unglaub, F., Wright, N.E., Zeng, W.Y., Paz-Yepes, J., Brahamsha, B., et al. (2014) Broad-host-range vector system for synthetic biology and biotechnology in cyanobacteria. *Nucleic Acids Research* **42**:.

Thomas, D.J., Avenson, T.J., Thomas, J.B., and Herbert, S.K. (1998) A Cyanobacterium Lacking Iron Superoxide Dismutase Is Sensitized to Oxidative Stress Induced with Methyl Viologen but Is Not Sensitized to Oxidative Stress Induced with Norflurazon1. *Plant physiology* **116**: 1593–1602.

Welsh, E.A., Liberton, M., Stöckel, J., Loh, T., Elvitigala, T., Wang, C., et al. (2008) The genome of Cyanothece 51142, a unicellular diazotrophic cyanobacterium important in the marine nitrogen cycle. *Proceedings of the National Academy of Sciences of the USA* **105**: 15094–9.

Xu, T., Qin, S., Hu, Y., Song, Z., Ying, J., Li, P., et al. (2016) Whole genomic DNA sequencing and comparative genomic analysis of Arthrospira platensis: High genome plasticity and genetic diversity. *DNA Research* **23**: 325–338.

Yerrapragada, S., Shukla, A., Hallsworth-Pepin, K., Choi, K., Wollam, A., Clifton, S., et al. (2015) Extreme Sensory Complexity Encoded in the 10-Megabase Draft Genome Sequence of the Chromatically Acclimating Cyanobacterium Tolypothrix sp. PCC 7601. *Genome Announcements*.

Zhao, W., Guo, Q., and Zhao, J. (2007) A membrane-associated Mn-superoxide dismutase protects the photosynthetic apparatus and nitrogenase from oxidative damage in the cyanobacterium Anabaena sp. PCC 7120. *Plant and Cell Physiology* **48**: 563–72.
